# Selective detection of DABCO using a supramolecular interconversion as fluorescence reporter

**DOI:** 10.3762/bjoc.15.137

**Published:** 2019-06-21

**Authors:** Indrajit Paul, Debabrata Samanta, Sudhakar Gaikwad, Michael Schmittel

**Affiliations:** 1 Center of Micro and Nanochemistry and Engineering, Organische Chemie I, Universität Siegen, Adolf-Reichwein-Str. 2, D-57068 Siegen, Germany

**Keywords:** copper, detection, fluorescence, interconversion, macrocycles, self-assembly, self-sorting, zinc porphyrin

## Abstract

The quantitative double self-sorting between the three-component rectangle [Cu_4_(**1**)_2_(**2**)_2_]^4+^ and the four-component sandwich complex [Cu_2_(**1**)(**2**)(**4**)]^2+^ is triggered by inclusion and release of DABCO (**4**). The fully reversible and clean switching between two multicomponent supramolecular architectures can be monitored by fluorescence changes at the zinc porphyrin sites. The structural changes are accompanied by a huge spatial contraction/expansion of the zinc porphyrin–zinc porphyrin distances that change from 31.2/38.8 Å to 6.6 Å and back. The supramolecular interconversion was used for the highly selective detection of DABCO in a mixture of other similar compounds.

## Introduction

Since dynamic multicomponent supramolecular structures are nowadays abundant [1–2], the weak intercomponent binding [3–9] is often instrumentalized for supramolecular transformations [10], but rarely exploited strategically for specific functions. Elegant examples for the utility of dynamic interactions, in particular metal–ligand coordination, are thermally driven supramolecular devices, such as ball bearings [11–12], crank engines [13], rotors [14–17] and oscillators [18]. Recent work from our group [19] has strived for combining metallosupramolecular transformation(s) [20] with the creation of emergent functions, for instance by presenting three-state catalytic machinery with up and down regulation [21] or for networking catalytic machinery [22].

In contrast, the actual contribution seeks to explore the utility of supramolecular rearrangements [23] for sensing and detection, in particular with an emphasis on high selectivity. As a notable example of the latter category, Nitschke recently reported the guest-induced transformation of porphyrin edge capsules to cone-shaped inclusion complexes depending on the presence of C_60_/C_70_, however, a process that was not selective for one of the guests [24]. A spectacular case of guest sensing, but not guest-induced recognition, was demonstrated by Clever in a supramolecular cage-to-cage conversion that allowed detection of the product by shape recognition [25]. Unfortunately, the cage-to-cage transformation proved to be rather slow. Schalley used the addition of both, guests and hosts, to stimulate a cascaded folding of cucurbit[7,8]uril pseudorotaxanes [26]. Neither of the above examples was demonstrated to be reversible after removing the guest. This compilation of remarkable results already indicates that guest-induced supramolecular transformations are not yet explored to their full potential.

**Figure 1:**
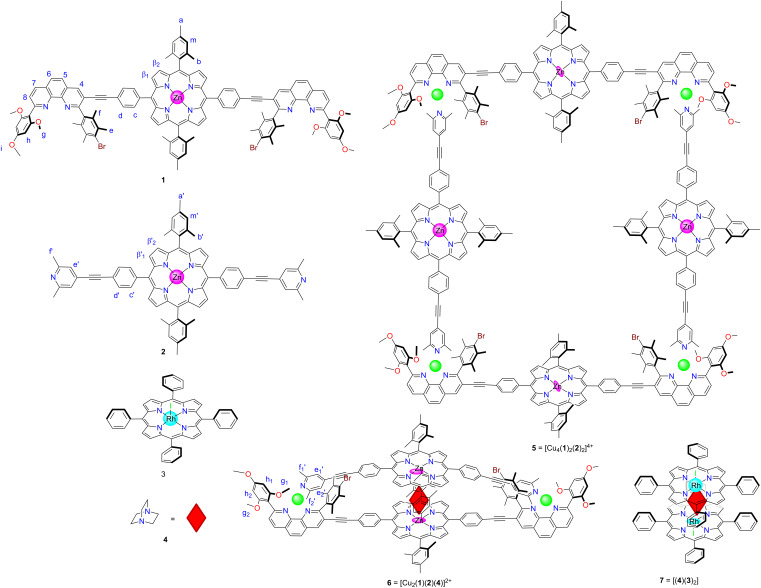
Molecular structures of ligands **1**, **2**, **3**, and **4** and of the resulting products, i.e., rectangle **5**, sandwich **6** and rhodium porphyrin dimer **7**.

**Scheme 1:**
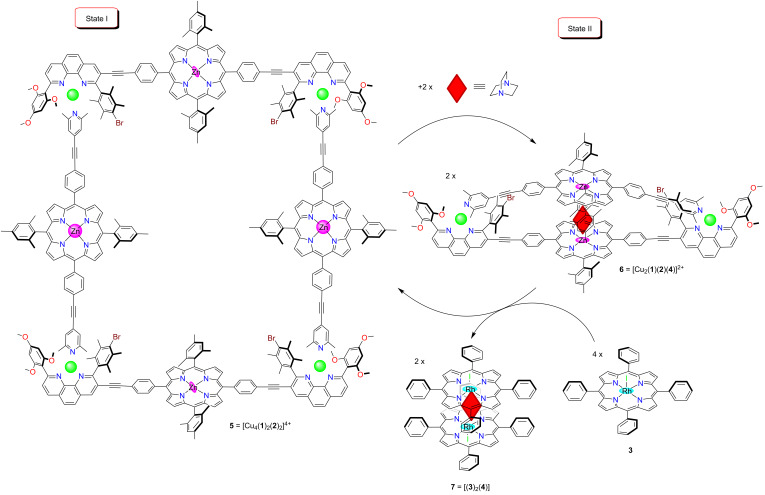
Guest addition/removal and reversible interconversion between supramolecular architectures.

Herein, we will present the formation of self-assembled three to four-component supramolecules, such as rectangle **5** and sandwich **6** (Figure 1), as well as their responsiveness to a variety of potential guests. Despite the topological simplicity of the assemblies involved, their multicomponent arrangements require perfect heteroleptic control. Conceptually, the process shown in Scheme 1 is a dual-state supramolecular transformation driven by addition/removal of DABCO (**4**) and it requires a transition between completive vs incomplete self-sorting [27–28]. The fact that DABCO (**4**) exclusively drives the supramolecular interconversion was further developed into a fluorescent reporter system [29–31] with high selectivity.

## Results and Discussion

**Scheme 2:**
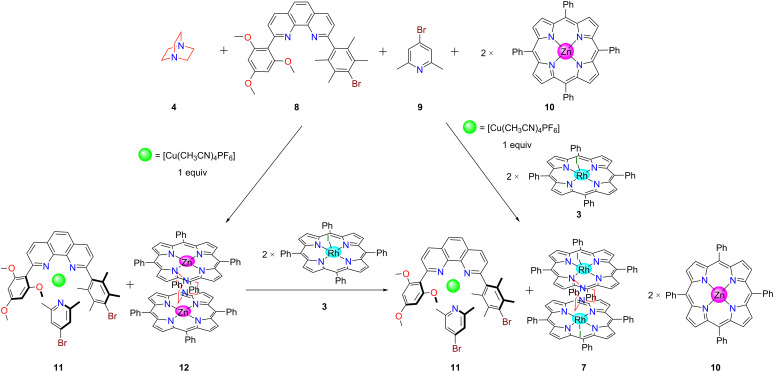
Completive and incomplete self-sorting in presence of copper(I) and rhodium complex **3**.

To realize the aspired switching protocol, we have tested metal-ion and guest-dependent completive and incomplete self-sorting scenarios [32] by mixing ligands **4**, **8**, **9**, **10** and [Cu(CH_3_CN)_4_]PF_6_ in a ratio of 1:1:1:2:1 (Scheme 2). Ligand **8** was equipped with a trimethoxyphenyl group to furnish a fourth coordination to the copper(I) center and a sterically crowded duryl group to prevent homoleptic complexation, while lutidine **9** was selected to strengthen the HETPYP-I [33] (HETeroleptic PYridine and Phenanthroline complexation) coordination. In this setting, the binary complex **12** = [(**4**)(**10**)_2_] and the heteroleptic metal complex **11** = [Cu(**8**)(**9**)]^+^ quantitatively formed side by side in a two-fold completive self-sorting. Lutidine **9** has a higher binding preference towards the copper phenanthroline [Cu(**8**)]^+^ (log *K*_(_**_9_**_)·[Cu(_**_8_**_)]_+ = 4.60 ± 0.21, Supporting Information File 1, Figure S24) than towards zinc porphyrin **10** (log *K*_(_**_9_**_)(_**_10_**_)_ = 1.82 ± 0.21) [34] due to its bulky α-methyl groups. Therefore, in the self-sorting zinc porphyrin **10** prefers to form the stable sandwich complex **12** with DABCO [35–36] at log β_[(_**_4_**_)(_**_10_**_)2]_ = 7.20 ± 0.15 (Supporting Information File 1, Figure S21) to satisfy maximum site occupancy.

Upon the addition of 2 equiv of rhodium porphyrin **3** [37], DABCO was selectively removed from complex **12** [38] affording the sandwich complex **7** = [(**3**)_2_(**4**)] leaving complex **11** untouched and liberating two equiv of **10** (incomplete self-sorting, Scheme 2). This phenomenon is readily explained considering the stronger binding of rhodium porphyrin **3** to DABCO (Δlog β = 2.40) compared with zinc porphyrin (Supporting Information File 1, Figure S22).

With this ligand shuffling in mind, we wanted to probe the guest-induced double self-sorting depicted in Scheme 1. Therefore, ligands **1** and **2** were synthesized by a palladium-catalyzed Sonogashira coupling reaction (Supporting Information File 1). All compounds were fully characterized by ^1^H NMR, ^1^H,^1^H-COSY, UV–vis, ESIMS and elemental analysis (Supporting Information File 1).

**Figure 2:**
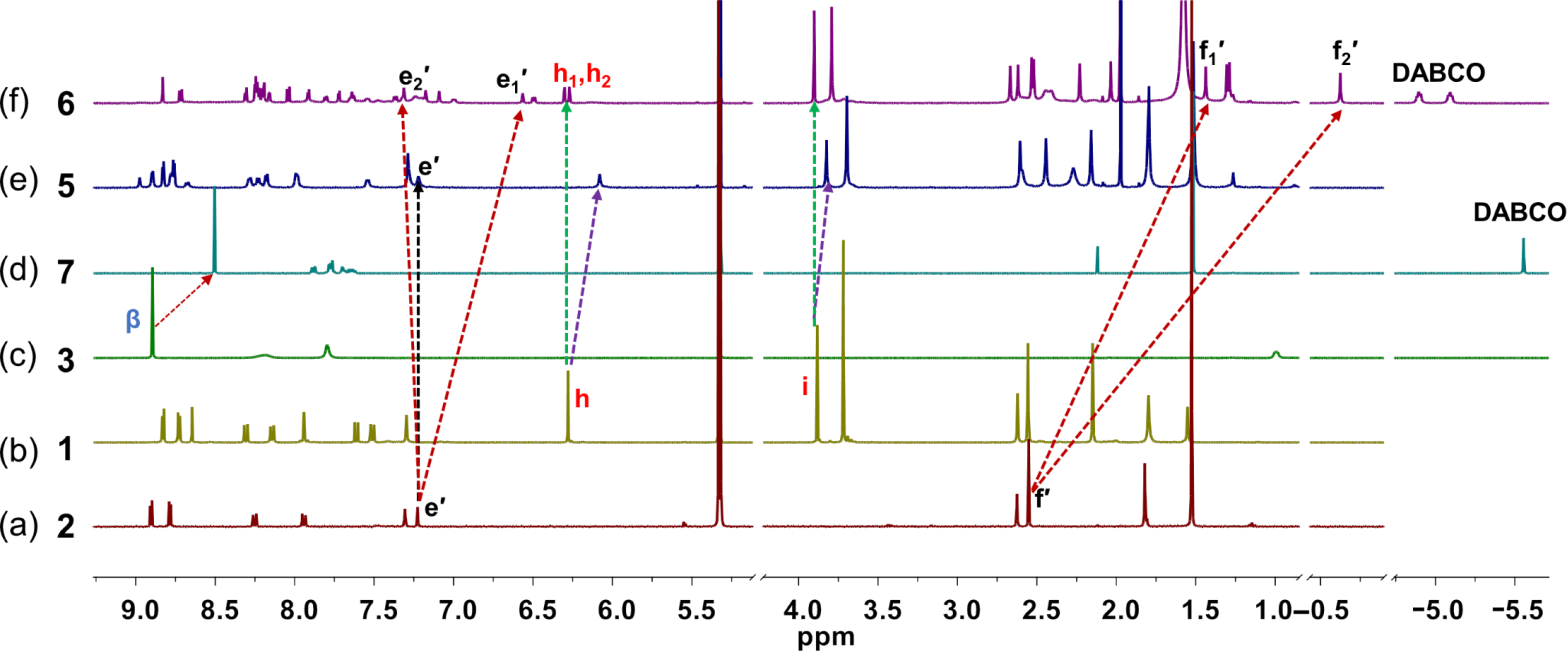
Comparison of partial ^1^H NMR spectra (400 MHz, CD_2_Cl_2_, 298 K) of (a) ligand **2**; (b) ligand **1**; (c) porphyrin **3**; (d) complex **7** = [(**3**)_2_(**4**)]; (e) rectangle **5** = [Cu_4_(**1**)_2_(**2**)_2_]^4+^]; (f) sandwich complex **6** = [Cu_2_(**1**)(**2**)(**4**)]^2+^.

Subsequently, we prepared the supramolecular rectangle **5** and sandwich complex **6**. At first, ligands **1**, **2**, and [Cu(CH_3_CN)_4_]PF_6_ (1:1:2) were mixed in CD_2_Cl_2_ immediately giving rise to rectangle **5** at room temperature. The clear red complex was characterized by ESIMS, ^1^H NMR, ^1^H,^1^H COSY, UV–vis and by elemental analysis (Supporting Information File 1). The ESIMS exhibited a single peak at *m*/*z* = 1534.5 (Supporting Information File 1, Figure S19) representing **5** = [Cu_4_(**1**)_2_(**2**)_2_]^4+^, constituting strong evidence that **5** is the sole product of this particular reaction. This notion was further ascertained by the ^1^H DOSY NMR (Supporting Information File 1, Figure S29) showing a single species with a diffusion coefficient of 2.43 × 10^−10^ m^2^s^−1^. The thus derived molecular radius of 21.7 Å is in very good agreement with the computed *r* = 21.5 Å (DFT, see Supporting Information File 1, Figure S31). Rectangle **5** was also characterized through the expected ^1^H NMR pattern in particular as the signal of proton h-H in ligand **1** is shifted diagnostically from 6.28 to 6.08 ppm due to the shielding of the lutidine unit by the π-system of ligand **2** (Figure 2).

Self-assembly in a similar manner using [Cu(CH_3_CN)_4_]PF_6_ and ligands **1**, **2** and **4** (2:1:1:1) afforded complex **6** = [Cu_2_(**1**)(**2**)(**4**)]^2+^ as the exclusive product at room temperature. A single diffusion coefficient in the ^1^H DOSY NMR (*D* = 4.40 × 10^−10^ m^2^s^−1^) as well as a single set of signals in the ^1^H NMR spectrum provided evidence of high purity. The experimental radius of 12.0 Å reflects the computed radius of the largely contracted aggregate (*r* = 12.3 Å). As seen in the ^1^H NMR, the lutidine unit of ligand **2** is split into two sets with proton e_1_’-H appearing at 6.56 ppm and e_2_’-H emerging at 7.32 ppm. The explicit upfield shift of proton e_1_’-H is due to shielding by the duryl group of the phenanthroline and very similar to the one experienced by the methyl protons (f_2_’-H) that are diagnostically shifted upfield to −0.63 ppm. Proton h-H is equally split into two sets reflecting the strong coordination of one methoxy group at an unsymmetrically coordinated copper(I) center. Similar to model system **12**, sandwich complex **6** experiences a strong upfield shift of the DABCO protons but now these protons are split into two sets at −4.89 and −5.09 ppm which clearly supports the formation of a hetero bisporphyrin sandwich (Figure 2). The ESIMS spectrum with its peak at *m/z* = 1589.4 is in line with the integrity of complex **6**. In sum, the clean formation of complexes **5** and **6** provides a reliable base for the elaboration of completive and incomplete double self-sorted guest-induced structural rearrangements.

**Figure 3:**
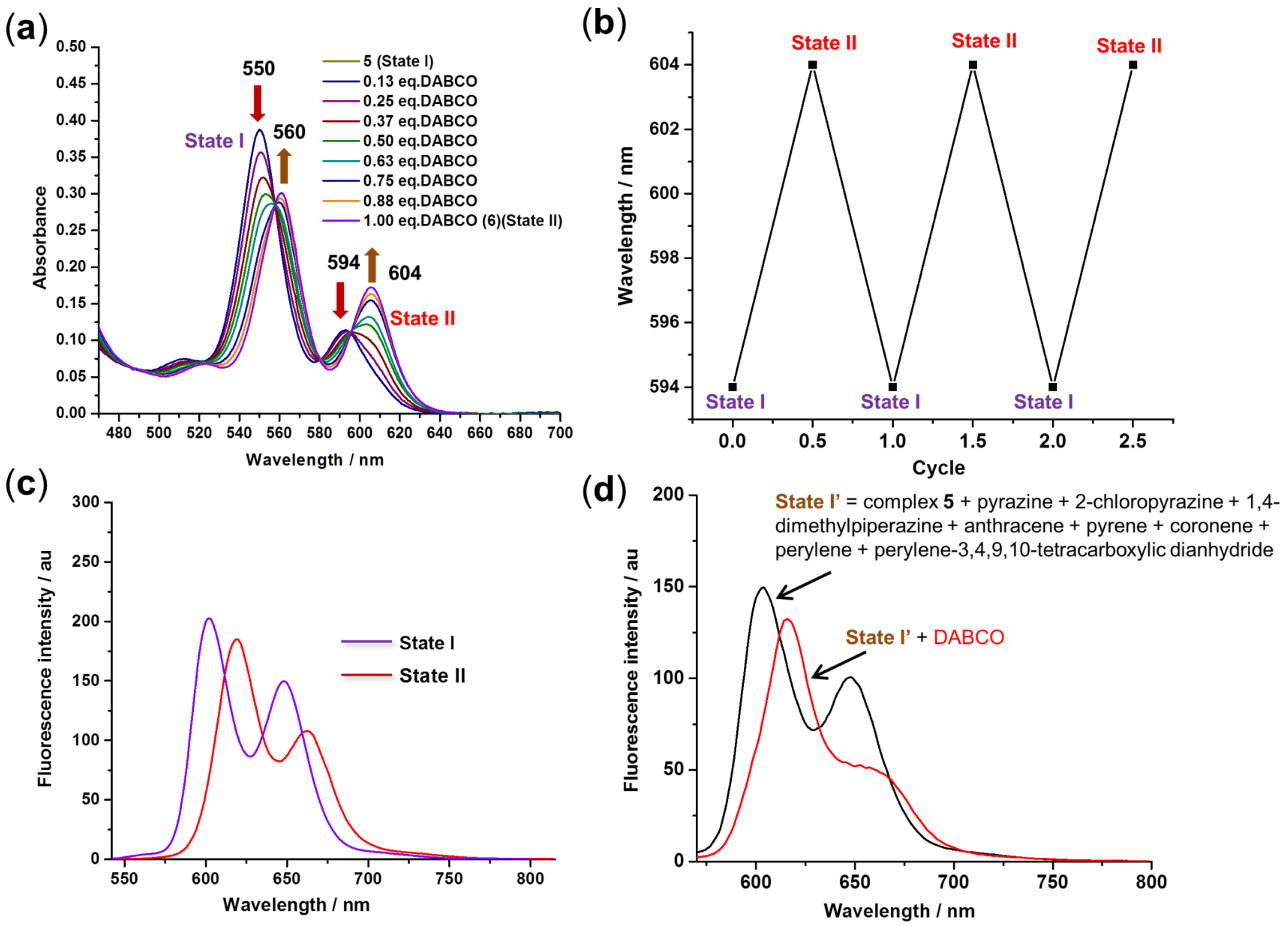
(a) UV–vis titration of rectangle **5** (2.98 μM) with DABCO (**4**); (b) several reversible cycles of interconversion monitored at λ = 594 and 604 nm; (c) emission spectra of states I and II (λ_exc_ = 557 nm); (d) emission spectra of **5** (λ_exc_ = 557 nm) after adding various potential guests.

In order to verify the forward conversion shown in Scheme 1, ligands **1** and **2** as well as [Cu(CH_3_CN)_4_]PF_6_ were mixed at a 1:1:2 ratio in CD_2_Cl_2_ to afford rectangle **5** (state I), as confirmed by ^1^H NMR. The rectangle furthermore exhibits (Figure 3a,c) diagnostic absorption bands at λ = 550 and 594 nm in dichloromethane (Q-band) and an emission at λ = 602 nm (excited at λ = 557 nm; isosbestic point of conversion **5** to **6**). When 1 equiv of DABCO (**4**) was added at room temperature, the deep red color of complex **5** immediately changed to greenish, furnishing the sandwich complex **6** (state II). As expected from the independently prepared sample, the ^1^H NMR shows two sets of DABCO protons at a 1:1 ratio and agreement with the full ^1^H NMR signature of **6**. The guest-induced conversion was further validated by a UV–vis titration. Upon the addition of DABCO the Q-band absorptions of **5** at λ = 550 and 594 nm shifted to λ = 560 and 604 nm which is expected for the N_DABCO_ → zinc porphyrin coordination [39–40]. Equally, the emission wavelength changes by addition of DABCO. Figure 3c nicely illustrates the shift of the emission band from λ = 602 → 618 nm (λ_exc_ = 557 nm) for the conversion of complex **5** → **6** illustrating that DABCO inclusion into the porphyrinic sandwich entails a shift of 16 nm. Finally, a single set of ^1^H DOSY confirms the successful rearrangement.

To probe the selectivity of the guest-induced transformation of **5**, the structure interconversion was tested with other potential guests, using fluorescence and ^1^H NMR spectroscopy. In the absence of any external ligand, the rectangle **5** shows its typical fluorescence at λ = 602 nm. Ligands, such as pyrazine, 2-chloropyrazine, 1,4-dimethylpiperazine, anthracene, pyrene, coronene, perylene, and perylene-3,4,9,10-tetracarboxylic dianhydride were compared to DABCO. Only in presence of DABCO the fluorescence maximum was shifted to λ = 618 nm along with the color changing from red to green. As displayed in Figure 3d, the results demonstrate that the emission doesn’t change in wavelength with any ligand except DABCO.

The high selectivity of architecture **5** towards DABCO was attributed to factors such as binding strength of the ditopic ligands and minimum steric repulsion. For instance, pyrazine creates notable repulsive interactions of the α-H towards the zinc porphyrin ring in a sandwich complex. Apparently, stability gains through π–π stacking in the sandwich with pyrene, coronene, etc. are not strong enough to compensate for the strain in [Cu_2_(**1**)(**2**)(guest)]^2+^. Encouraged by this finding, we decided to probe the selectivity for DABCO in the presence of a mixture of all ligands (all ligands used at the same molar amount). The emission is the same as that in Figure 3c for DABCO alone demonstrating that DABCO is cleanly selected even in such complex mixtures. Thus, DABCO is a highly selective trigger for the structural rearrangement of rectangle **5** to sandwich complex **6**.

**Scheme 3:**
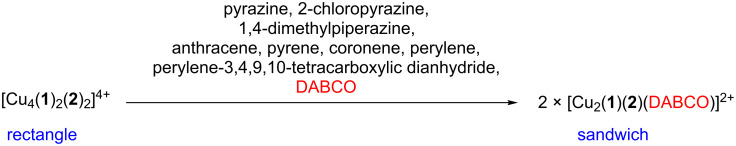
The high selectivity for DABCO in the transformation.

**Figure 4:**
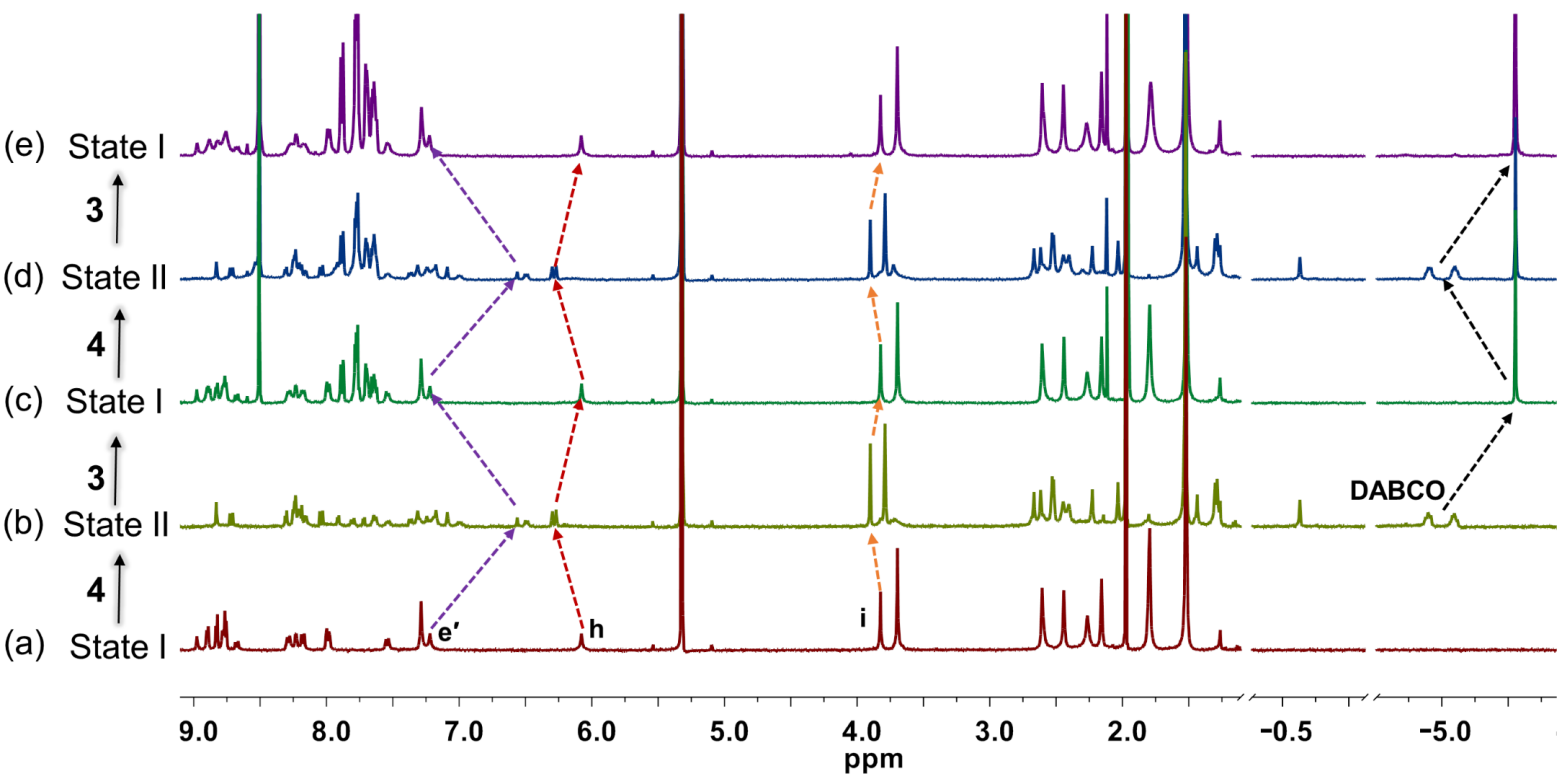
Partial spectra (400 MHz, CD_2_Cl_2_, 298 K) showing the reversible switching between rectangle and sandwich complexes over 2.0 cycles; (a) after mixing of [Cu(CH_3_CN)_4_]PF_6_, **1**, and **2** in 2:1:1 ratio, furnishing rectangle **5** (state I); (b) after adding 2.0 equiv of DABCO, furnishing sandwich complex **6** (state II); (c) after addition of 4.0 equiv of rhodium porphyrin **3**, leading to formation of rectangle **5** and rhodium porphyrin dimer **7**. (d) Further addition of 2.0 equiv of DABCO furnishes state II; (e) finally, addition of 4.0 equiv of rhodium porphyrin **3** recovered rectangle **5** along with 4.0 equiv of complex **7**.

Finally, we tested the reversibility of the system by addition and removal of DABCO using rhodium porphyrin **3** as scavenger of DABCO. In line with the results of the model self-sorting scenarios in Scheme 2, the system turned out to be fully reversible without loss (Scheme 3). For example, state II (= 2 × [Cu_2_(**1**)(**2**)(**4**)]^2+^) was easily converted to state I by addition of 4 equiv of **3** and state II was again regained by addition of 2 equiv of DABCO. Multiple cycles of state I → state II transformations were established through ^1^H NMR (Figure 4) and UV–vis spectra (Figure 3b).

DFT (B3LYP/6-31G(d)) calculations on rectangle **5** and sandwich complex **6** allow the modeling of the supramolecular architecture and provide some structural insights. The DFT computations demonstrate that the sandwich complex is quite strained and structurally distorted to a spiral shape (Supporting Information File 1, Figures S31 and S32).

## Conclusion

In conclusion we demonstrated three cycles of the fully reversible DABCO-induced structural rearrangement between multicomponent architectures **5** and **6**. The multiple, clean and quantitative interconversion is the result of a delicate double self-sorted transformation requiring orthogonality of two heteroleptic complexation motifs (HETPYP-I and hetero-sandwich complexation at DABCO). Within a selected library of binding guests, DABCO is the only one effecting the interconversion. Due to the fact, that the interconversion is accompanied by a diagnostic change in the fluorescence spectra, the present system represents a supramolecular reporter for the selective detection of DABCO. It is thus a rare example of DABCO sensing by luminescence [41].

## Supporting Information

File 1 Experimental details and characterization data.
