# Supplementary material for: Selective detection of DABCO using a supramolecular interconversion as fluorescence reporter
Source: Beilstein J Org Chem. 2019 Jun 21;15:1371–8. doi: 10.3762/bjoc.15.137 (PMC6604717; doi:10.3762/bjoc.15.137)
Supplement: File 1 — Experimental details and characterization data. [file Beilstein_J_Org_Chem-15-1371-s001.pdf]

BEILSTEIN JOURNAL OF ORGANIC CHEMISTRY

Supporting Information

for

Selective detection of DABCO using a supramolecular interconversion as fluorescence reporter

Indrajit Paul, Debabrata Samanta, Sudhakar Gaikwad and Michael Schmittel

Beilstein J. Org. Chem. **2019,** *15,* 1371–1378. doi:10.3762/bjoc.15.137

Experimental details and characterization data

License and Terms: This is a supporting information file under the terms of the Creative Commons Attribution License (http://creativecommons.org/licenses/by/4.0). Please note that the reuse, redistribution and reproduction in particular requires that the authors and source are credited.
The license is subject to the *Beilstein Journal of Organic Chemistry* terms and conditions: (https://www.beilstein-journals.org/bjoc)

Table of Contents

1. Synthesis (general remarks) S2
2. Synthesis and characterization of ligands S5
3. Synthesis and characterization of model complexes S7
4. Synthesis and characterization of supramolecular architectures S9
5. Model studies S10
6. Selectivity and guest-induced structural rearrangement S12
7. NMR spectra: ^{1}H NMR, ^{13}C NMR, ^{1}H,^{1}H COSY S13
8. ESIMS spectra S19
9. UV–vis data S21
10. Fluorescence data S25
11. DOSY NMR S26
12. Calculated structures S27
13. References S28

1. Synthesis (general remarks)

1.1 General remarks on instrumentation and chemicals

All solvents were dried by distillation prior to use while commercial reactants (**4**, **9**, **10**) were utilized without any further purification. A Bruker Avance (400 MHz) spectrometer was employed to measure ^{1}H and ^{13}C NMR spectra using a deuterated solvent as the lock and residual protiated solvent as internal reference ($CDCl_3$: δ_H 7.26 ppm, δ_C 77.0 ppm; CD_2Cl_2: δ_H 5.32 ppm, δ_C 53.8 ppm, THF-d_8: δ_H 1.72 ppm, 3.58 ppm, δ_C 25.3 ppm, 67.2 ppm). The following abbreviations were used to define NMR peak patterns: s = singlet, d = doublet, t = triplet, br = broad, m = multiplet. The coupling constants are given in hertz (Hz) and, wherever possible, assignment of protons is made. The carbons in the molecular skeletons are mostly not numbered following the IUPAC nomenclature rules; it was exclusively done for assigning NMR signals. All electrospray ionization (ESIMS) spectra were recorded on a Thermo-Quest LCQ deca and the theoretical isotopic distributions of the mass signals were calculated using the IsoPro 3.0 software. Melting points of compounds were measured on a Büchi 510 instrument and are not corrected. Infrared spectra were recorded on a Varian 1000 FTIR instrument. Elemental analysis was performed using the EA-3000 CHNS analyzer. UV–vis spectra were recorded on a Cary Win 50 (298 K) spectrometer. Binding constants were determined through

UV–vis titrations assuming a 1:1 binding scheme or with the SPECFIT/32™ global analysis system by Spectrum Software Associates (Marlborough, MA). Column chromatography was performed either on silica gel (60–400 mesh) or neutral alumina (Fluka, 0.05–0.15 mm, Brockmann Activity 1). Merck silica gel (60 F254) or neutral alumina (150 F254) sheets were used for thin layer chromatography (TLC). All metal–ligand complexes were prepared directly in the NMR tube using CD_2Cl_2 as solvent. Compounds **8**,[1] **13**,[1] **14**,[2] and **15**[3] were synthesized according to literature known procedures.

1.2 General remarks on synthetic schemes

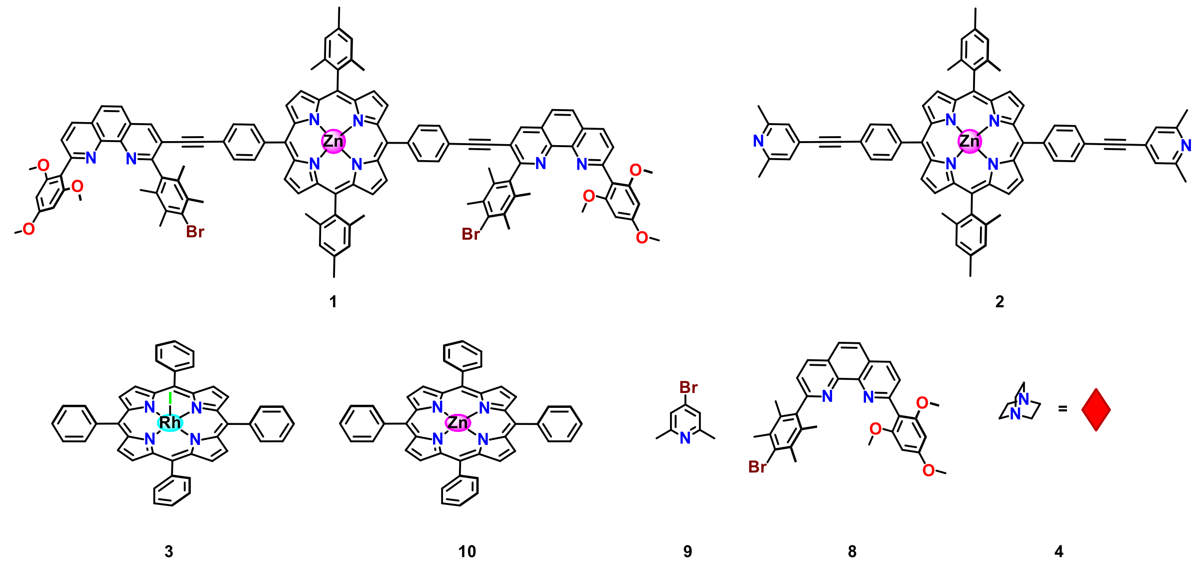

Chart S1: Ligands used in the present study.

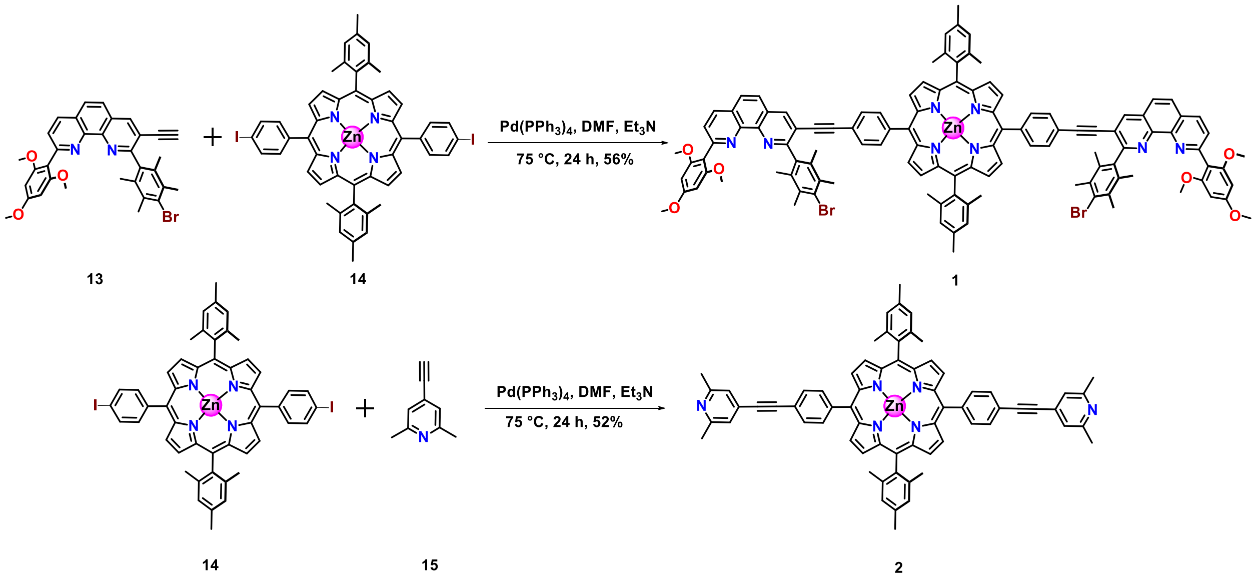

Scheme S1. Synthetic scheme used in the preparation of ligands **1** and **2.**

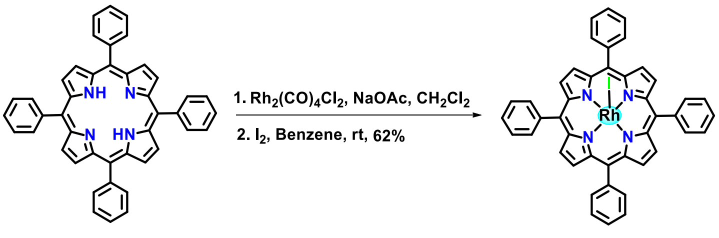

8 3

Scheme S2. Synthetic scheme used in the preparation of porphyrin **3**.

2. Synthesis and characterization of ligands

Synthesis of ligand 1

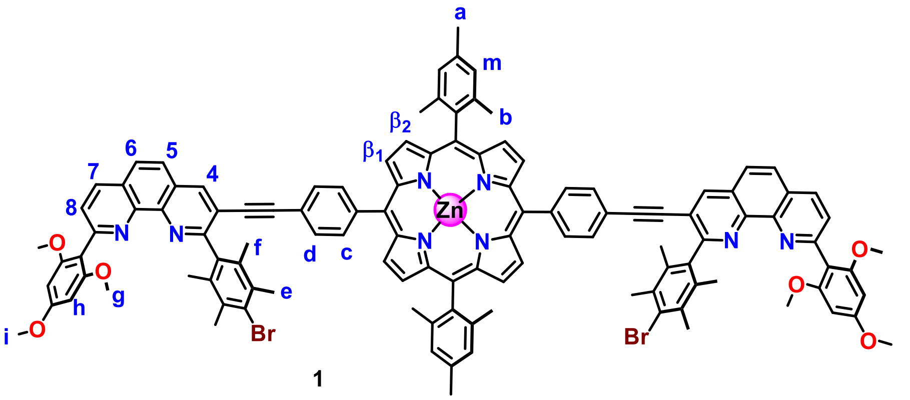

1

Under an argon atmosphere porphyrin **14**[2] (200 mg, 197 µmol) and phenanthroline **13**[1] (263 mg, 493 µmol) were dissolved in 15 mL of dry DMF and 15 mL of trimethylamine. Subsequently, the solution was deaerated by bubbling nitrogen through the solution for 1 h. Then the solution was charged with $Pd(PPh_3)_4$ (23.0 mg, 19.7 µmol) and heated at 75 °C for 24 h. The resulting solution was evaporated to dryness and the crude product was purified by column chromatography (silica gel, DCM, R_f = 0.4) furnishing 210 mg of **1** as red solid (110 µmol, 56%); **Mp**: >300 °C. **IR (KBr):** $\tilde{\nu}$ = 536, 576, 602, 622, 753, 815, 876, 903, 938, 992, 1119, 1191, 1255, 1285, 1347, 1390, 1436, 1473, 1518, 1605, 2191, 2201, 2362, 2891, 2858, 3077 cm^{-1}. **^{1}H NMR (400 MHz, CD_2Cl_2):** δ = 1.79 (s, 12H, b-H), 2.14 (s, 12H, e-H), 2.55 (s, 12H, f-H), 2.62 (s, 6H, a-H), 3.71 (s, 12H, g-H), 3.88 (s, 6H, i-H), 6.28 (s, 4H, h-H), 7.29 (s, 4H, m-H), 7.51 (d, ^3J = 8.0 Hz, 4H, c-H), 7.61 (d, ^3J = 8.0 Hz, 2H, 8-H), 7.93 (d, ^3J = 8.4 Hz, 2H, 5/6-H), 7.95 (d, ^3J = 8.4 Hz, 2H, 6/5-H), 8.14 (d, ^3J = 8.0 Hz, 4H, d-H), 8.83 (d, ^3J = 8.0 Hz, 2H, 7-H), 8.65 (s, 2H, 4-H), 8.72 (d, ^3J = 4.8 Hz, 4H, β_2-H), 8.82 (d, ^3J = 4.8 Hz, 4H, β_1-H) ppm. **^{13}C NMR (100 MHz, CD_2Cl_2):** δ = 18.7, 21.1, 21.5, 21.7, 55.8, 56.3, 87.8, 91.2, 95.7, 113.1, 119.6, 119.7, 120.1, 122.0, 126.0, 127.1, 127.4, 127.6, 127.9, 128.3, 129.3, 129.9, 131.1, 132.3, 133.9, 134.3, 134.9, 135.8, 137.9, 139.0, 139.4, 139.4, 139.9, 143.9, 145.4, 146.3, 150.01, 150.2, 156.1, 159.3, 162.0, 162.6, 162.0 ppm. **ESI-MS:** *m/z* (%) = 1921.4 (50) [**1**+H]$^+$, 961.8 (100) [**1**+2H]$^{2+}$. **Elemental analysis:** Calcd. for $C_{116}H_{94}Br_2N_8O_6Zn$: C, 72.52; H, 4.93; N, 5.83. Found: C, 72.31; H, 5.12; N, 5.74.

Synthesis of Ligand 2:

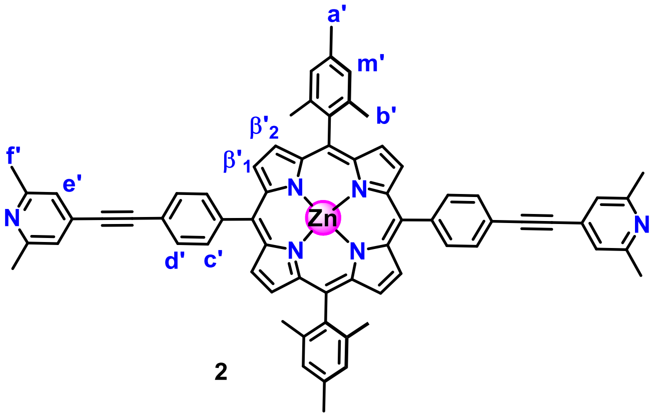

2

Porphyrin **14**[2] (100 mg, 98.5 µmol) and 4-ethynyl-2,6-dimethylpyridine (**15**[3], 32.3 mg, 246 µmol) were loaded into sealed tube. After the addition of dry triethylamine (10 mL) and dry DMF (10 mL), the mixture was deaerated by bubbling nitrogen through it for 30 min. Then, solid $Pd(PPh_3)_4$ (12 mg, 9.85 µmol) was added under a steady flow of nitrogen and the mixture stirred at 75 °C for 24 h. After completion, the solvents were evaporated under reduced pressure. The residue was dissolved in DCM (50 mL) and washed with water (100 mL). After drying over Na_2SO_4, the solvent was removed and the crude product was purified by column chromatography (SiO_2) using DCM as eluent to afford 52.0 mg (51.0 µmol, 52%) of the desired product **2** as a violet solid (silica gel, DCM, R_f = 0.5). **Mp:** >300 °C. **IR (KBr):** $\tilde{\nu}$ = 506, 616, 748, 790, 863, 989, 1038, 1094, 1246, 1390, 1469, 1581, 2155, 2221, 2896, 2955, 3013, 3058, cm^{-1}. **^{1}H NMR (400 MHz, CD_2Cl_2):** δ = 1.82 (s, 12H, b'-H), 2.55 (s, 12H, f'-H), 2.62 (s, 6H, a'-H), 7.22 (s, 4H, e'-H), 7.30 (s, 4H, m'-H), 7.93 (d, ^3J = 8.0 Hz, 4H, c'-H), 8.24 (d, ^3J = 8.0 Hz, 4H, d'-H), 8.74 (d, ^3J = 4.8 Hz, 4H, β'$_2$-H), 8.90 (d, ^3J = 4.8 Hz, 4H, β'$_1$-H) ppm. **^{13}C NMR (100 MHz, CD_2Cl_2):** δ = 21.5, 21.7, 24.5, 88.4, 92.8, 119.7, 119.9, 121.9, 122.2, 127.9, 130.4, 131.2, 131.8, 132.4, 134.9, 138.0, 139.2, 139.4, 144.0, 150.1, 150.3, 158.4 ppm. **ESI-MS:** *m/z* (%) = 1019.7 (100) [**2**+H]$^+$; **Elemental analysis:** Calcd. for $C_{68}H_{54}N_6Zn \bullet H_2O$: C, 78.64; H, 5.43; N, 8.09. Found C, 78.46; H, 5.37; N, 8.22.

Synthesis of porphyrin 3[4]

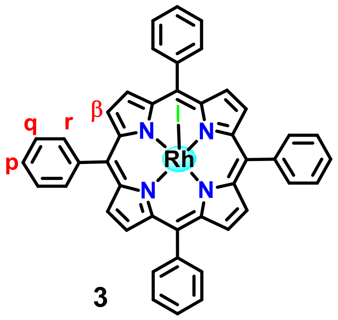

3

Under a nitrogen atmosphere, $Rh_2(CO)_4Cl_2$ (47.0 mg, 121 µmol) and 5,10,15,20-tetraphenyl-porphyrin (50.0 mg, 81.3 µmol) were dissolved in anhydrous CH_2Cl_2 (10 mL). After the addition of anhydrous NaOAc (198 mg, 2.42 mmol) stirring was continued for 45 min. Avoiding any exposure to air, the reaction mixture was concentrated under vacuum (5 min, to remove residual acetic acid), a new portion of CH_2Cl_2 (10 mL) was added, and the procedure was repeated. The reaction mixture was dissolved in dry benzene (10 mL) and I_2 (21.0 mg, 121 µmol) was added. The progress of oxidation was monitored by UV–vis analysis and TLC. As soon as the reaction was completed (45 min), the mixture was transferred to a silica column and the product was eluted with CH_2Cl_2. After evaporation under vacuum 42.0 mg (50.4 µmol, 62%) of reddish product **3** (silica gel, DCM, R_f = 0.4) were obtained. **Mp:** >300 °C. **IR (KBr):** $\tilde{\nu}$ = 3058, 3013, 2955, 2896, 1581, 1469, 1390, 1246, 1094, 1038, 989, 863, 790, 748, 616, 506 cm^{-1}. **^{1}H NMR (400 MHz, CD_2Cl_2):** δ = 7.74-7.84 (m, 12H, p-,r-H), 8.11-8.30 (m, 8H, q-H), 8.89 (s, 8H, β-H) ppm.

3. Synthesis and characterization of model complexes

General procedure: All solid compounds were placed in an NMR tube and then dissolved in CD_2Cl_2. Subsequently, the NMR spectra were recorded at 298 K.

Model complex 11 = [Cu(**8**)(**9**)]$^+$

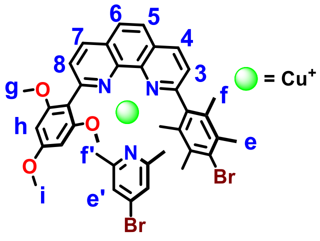

In an NMR tube, compound **8** (1.33 mg, 2.21 µmol), 4-bromo-2,6-dimethylpyridine (**9**, 412 µg, 2.21 µmol), and $[Cu(CH_3CN)_4]PF_6$ (825 µg, 2.21 µmol) were mixed in 500 µL of CD_2Cl_2 at room temperature. **Yield**: Quantitative. **^{1}H NMR (400 MHz, CD_2Cl_2):** δ = 1.94 (s, 6H, e-H), 2.17 (s, 6H, f'-H), 2.38 (s, 6H, f-H), 3.60 (s, 6H, g-H), 3.73 (s, 3H, i-H), 5.98 (s, 2H, h-H), 7.24 (s, 2H, e'-H), 7.88 (d, ^3J = 8.0 Hz, 1H, 3/8-H), 7.91 (d, ^3J = 8.0 Hz, 1H, 8/3-H), 8.14 (s, 2H, 5-,6-H), 8.60 (d, ^3J = 8.0 Hz, 1H, 4/7-H), 8.70 (d, ^3J = 8.0 Hz, 1H, 7/4-H) ppm. **ESI-MS:** *m/z* (%) = 853.8 (100) [Cu(**8**)(**9**)]$^+$.

Model complex 12 = [DABCO(**10**)$_2$]

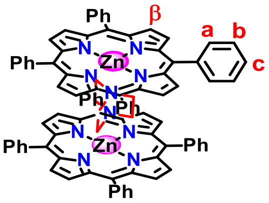

In an NMR tube DABCO (**4**, 150 µg, 1.34 µmol) and ZnTPP (**10**, 1.81 mg, 2.68 µmol) were mixed in 500 µL of CD_2Cl_2 at room temperature. **Yield**: Quantitative. **^{1}H NMR (400 MHz, CD_2Cl_2):** δ = −4.67 (s, 12H, CH_2-DABCO), 7.63-7.79 (m, 24H, a-,c-H), 7.86-8.09 (m, 16H, b-H), 8.67 (s, 16H, β-H) ppm.

Model complex 7 = [DABCO(**3**)$_2$]

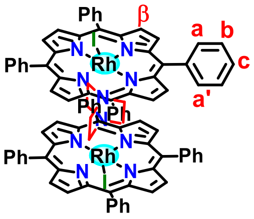

In an NMR tube DABCO (**4**, 110 µg, 0.982 µmol) and porphyrin **3** (1.65 mg, 1.96 µmol) were mixed in 500 µL of CD_2Cl_2 at room temperature. **Yield**: Quantitative. **^{1}H NMR (400 MHz, CD_2Cl_2):** δ = −5.55 (s, 12H, CH_2-DABCO), 7.61- 7.67 (m, 8H, a-/a'-H), 7.67-7.72 (m, 8H, a'-/a-H), 7.74-7.80 (m, 16H, b-H), 7.86-7.90 (m, 8H, c-H) ppm.

4. Synthesis and characterization of supramolecular architectures

Synthesis of complex 5 = [Cu_4(**1**)$_2$(**2**)$_2$]$^{4+}$

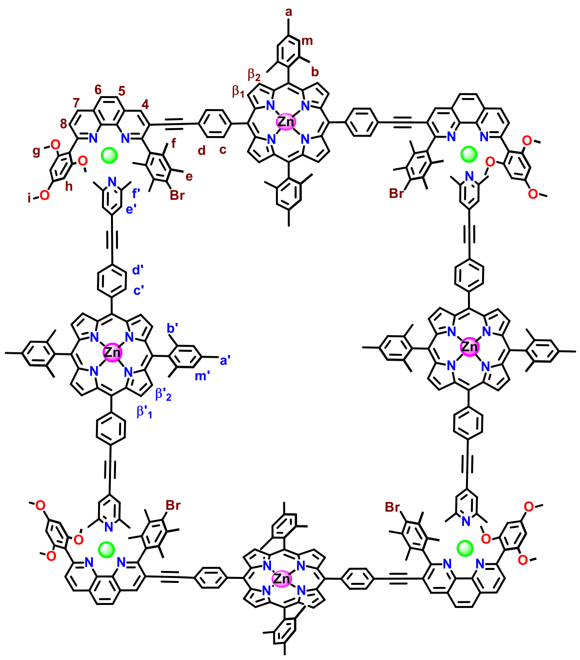

In an NMR tube, ligand **1** (911 µg, 0.474 µmol), ligand **2** (483 µg, 0.474 µmol), and [$Cu(CH_3CN)_4$]PF_6 (353 µg, 0.948 µmol) were dissolved in 500 µL of CD_2Cl_2. **Yield**: Quantitative. **^{1}H NMR (400 MHz, CD_2Cl_2):** δ = 1.79 (s, 24H, b-H), 1.80 (s, 24H, b'-H), 2.16 (s, 24H, e-H), 2.27 (s, 24H, f'-H), 2.44 (s, 24H, f-H), 2.59 (s, 12H, a'-H), 2.61 (s, 12H, a-H), 3.70 (s, 24H, g-H), 3.82 (s, 12H, i-H), 6.08 (s, 8H, h-H), 7.22 (s, 8H, e'-H), 7.29 (s, 16H, m-, m'-H), 7.54 (d, ^3J = 8.0 Hz, 8H, c-H), 7.96-7.80 (m, 12H, 8-, c'-H), 8.18 (d, ^3J = 8.0 Hz, 8H, d-H), 8.22 (d, ^3J = 8.4 Hz, 4 H, 5/6-H), 8.24 (d, ^3J = 8.4 Hz, 4H, 6/5-H), 8.28 (d, ^3J = 8.0 Hz, 8H, d'-H), 8.67 (d, ^3J = 8.0 Hz, 4H, 7-H), 8.76 (d, ^3J = 4.8 Hz, 8H, β_2-H), 8.78 (d, ^3J = 4.8 Hz, 8H, β'_2-H), 8.83 (d, ^3J = 4.8 Hz, 8H, β_1-H), 8.90 (d, ^3J = 4.8 Hz, 8H, β'_1-H), 8.98 (s, 4H, 4-H) ppm. **ESI-MS:** *m/z* (%) = 1534.5 (100) [Cu_4(**1**)$_2$(**2**)$_2$]$^{4+}$. **Elemental analysis:** Calcd for $C_{368}H_{296}Br_4Cu_4F_{24}N_{28}O_{12}P_4Zn_4 \bullet 2H_2O$: C, 65.44; H, 4.48; N, 5.81. Found C, 65.19; H, 4.56; N, 5.74.

Synthesis of complex 6 = $[Cu_2(\mathbf{1})(\mathbf{2})DABCO]^{2+}$

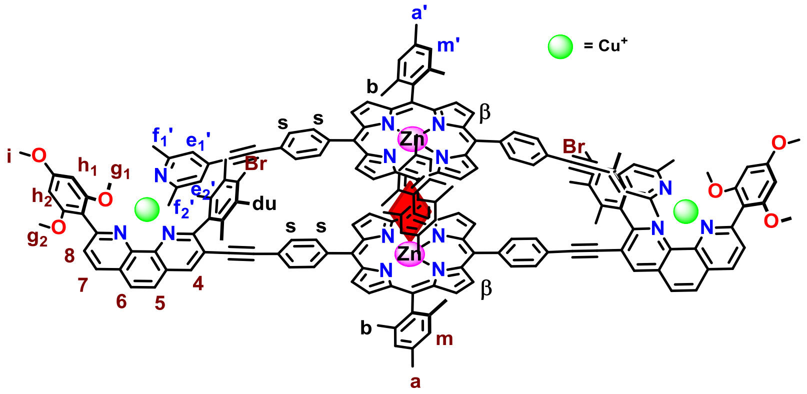

In an NMR tube, ligand **1** (911 µg, 0.474 µmol), ligand **2** (483 µg, 0.474 µmol), DABCO (**4**) (53.1 µg, 0.474 µmol) and $[Cu(CH_3CN)_4]PF_6$ (353 µg, 0.948 µmol) were dissolved in 500 µL of CD_2Cl_2. **Yield**: Quantitative; **^{1}H NMR (400 MHz, CD_2Cl_2):** δ = –[5.12-5.05] (m, 6H, CH_2, DABCO), –[4.92-4.85] (m, 6H, CH_2, DABCO), –0.63 (s, 6H, f_1'-/f_2'-H), 1.26 (s, 3H, b-H), 1.29 (br s, 6H, b-H), 1.30 (s, 3H, b-H), 1.44 (s, 6H, f_2'-/f_1'-H), 2.03 (s, 6H, a'-H), 2.23 (s, 6H, a-H), 2.40 (s, 6H, du-H), 2.45 (s, 6H, du-H), 2.52 (s, 6H, du-H), 2.53 (s, 6H, du-H), 2.62 (s, 6H, b-H), 2.67 (s, 6H, b-H), 3.78 (s, 6H, g_1-/g_2-H), 3.79 (s, 6H, g_2-/g_1-H), 3.90 (s, 6H, i-H), 6.26 (d, ^4J = 1.9 Hz, 2H, h_1-/h_2-H), 6.30 (d, ^4J = 1.9 Hz, 2H, h_2-/h_1-H), 6.49 (d, ^3J = 8.0 Hz, 2H, s-H), 6.56 (s, 2H, e_1'/e_2'-H), 6.99 (d, ^3J = 8.0 Hz, 2H, s-H), 7.09 (s, 2H, m-H), 7.17 (s, 2H, m-H), 7.19 (s, 2H, m'-H), 7.24 (s, 2H, m'-H), 7.30 (d, ^3J = 8.0 Hz, 2H, s-H), 7.32 (s, 2H, e_2'/e_1'-H), 7.36 (d, ^3J = 8.0 Hz, 2H, s-H), 7.54 (d, ^3J = 8.0 Hz, 2H, s-H), 7.62-7.66 (m, 4H, s-H), 7.71 (d, ^3J = 4.8 Hz, 2H, β-H), 7.80 (d, ^3J = 8.0 Hz, 2H, s-H), 7.91 (d, ^3J = 4.8 Hz, 2H, β-H), 8.04 (d, ^3J = 8.0 Hz, 2H, 8-H), 8.15 (d, ^3J = 4.8 Hz, 2H, β-H), 7.17-7.26 (m, 12H, [4β,5,6]-H), 8.30 (d, ^3J = 4.8 Hz, 2H, β-H), 8.71 (d, ^3J = 8.0 Hz, 2H, 7-H), 8.83 (s, 2H, 4-H) ppm; **ESI-MS:** *m/z* (%) = 1589.4 (100) $[Cu_2(\mathbf{1})(\mathbf{2})DABCO]^{2+}$. **Elemental analysis:** Calcd. for $C_{184}H_{148}Br_2Cu_2F_{12}N_{14}O_6P_2Zn_2 \bullet CH_3CN$: C, 65.71; H, 4.48; N, 6.18. Found: C, 65.38; H, 4.41; N, 6.32.

5. Model studies

Guest and metal-dependent two-fold completive self-sorting was tested by mixing ligand **8** (1.33 mg, 2.21 µmol), 4-bromo-2,6-dimethylpyridine (**9**, 412 µg, 2.21 µmol), ZnTPP (**10**, 3.00 mg, 4.41 µmol), DABCO (**4**, 248 µg, 2.21 µmol) and $[Cu(CH_3CN)_4]PF_6$ (825 µg, 2.21 µmol) in a ratio of 1:1:2:1:1 in 500 µL of CD_2Cl_2 at room temperature. The ^{1}H NMR spectrum was compared with those of the

individual ligands and complexes. Accordingly, **11** = [Cu(**8**)(**9**)]$^+$ and 12 = [DABCO(**10**)$_2$] were formed quantitatively.

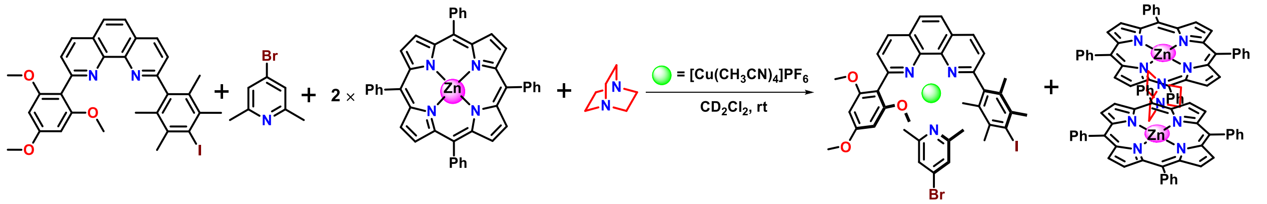

8 **9** **10** **4** **11** **12**

To the above reaction mixture rhodium porphyrin **3** (3.71 mg, 4.41 μmol) was added at room temperature. The ^{1}H NMR spectrum was compared with those of the individual NMRs attesting formation of complexes [Cu(**8**)(**9**)]$^+$, [DABCO(**3**)$_2$] and free ZnTPP. This study clearly indicates double self-sorting phenomena.

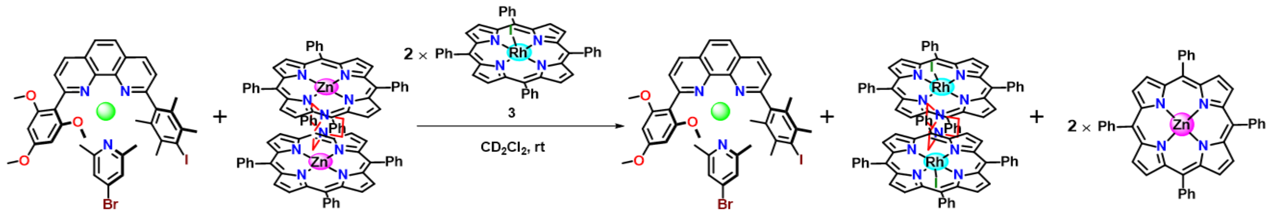

11 **12** **3** **11** **7** **10**

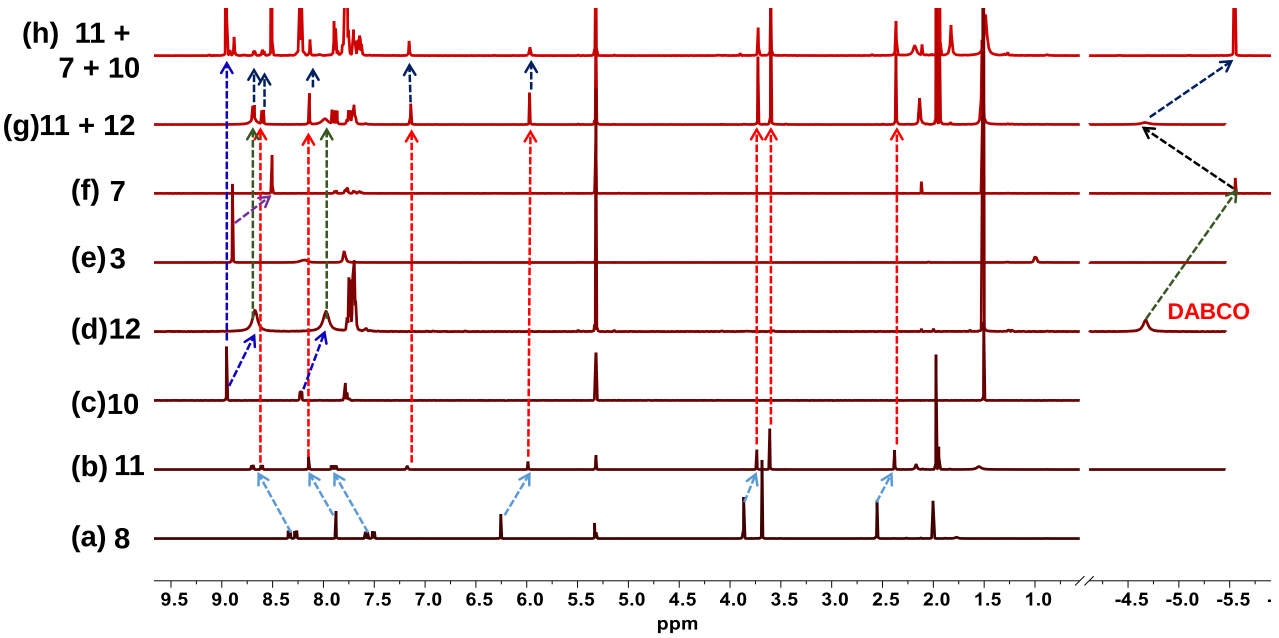

Figure S1. ^{1}H NMR (400 MHz, 298 K) of (a) ligand **8**; (b) complex **11** = [Cu(**8**)(**9**)]+; (c) ZnTPP (**10**) ; (d) complex **12** = [DABCO(**10**)$_2$]; (e) rhodium porphyrin **3**; (f) complex **7** = [DABCO(**3**)$_2$]; (g) formation of **11** and **12** after mixing of **8**, **9**, **10**, DABCO and [Cu(CH$_3$CN)$_4$]PF$_6$ in a ratio of 1:1:2:1; (h) formation of **11** and **7** as well as free porphyrin **10** after mixing of porphyrin **3** (2 equiv.) and mixture of **11** and **12**.

6. Selectivity and guest-induced structural rearrangement

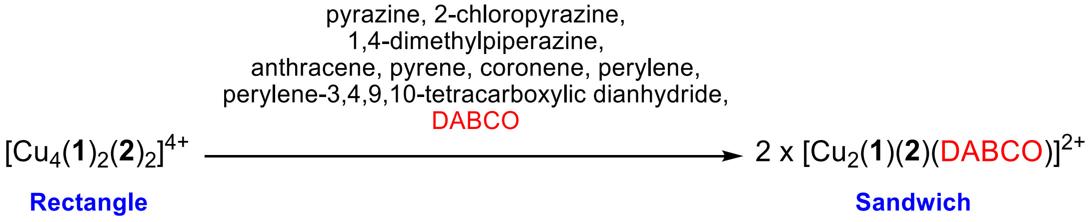

Guest selectivity and structural rearrangement was tested by mixing ligand **1** (736 µg, 0.383 µmol), ligand **2** (390 µg, 0.383 µmol), $[Cu(CH_3CN)_4]PF_6$ (284 µg, 0.766 µmol), pyrazine (30.0 µg, 0.383 µmol), 2-chloropyrazine (43.8 µg, 0.383 µmol), 1,4-dimethylpiperizine (43.7 µg, 0.383 µmol), anthracene (68.2 µg, 0.383 µmol), pyrene (77.4 µg, 0.383 µmol), coronene (115 µg, 0.383 µmol), perylene-3,4,9,10-tetracarboxylic dianhydride (150 µg, 0.383 µmol), and DABCO (42.8 µg, 0.383 µmol) in 500 µL of CD_2Cl_2 at room temperature followed by measurement of the ^{1}H NMR spectrum. The main part of the spectrum agreed with that of complex $[Cu_2(\mathbf{1})(\mathbf{2})DABCO]^{2+}$ with the rest of the signals representing free guests in solution.

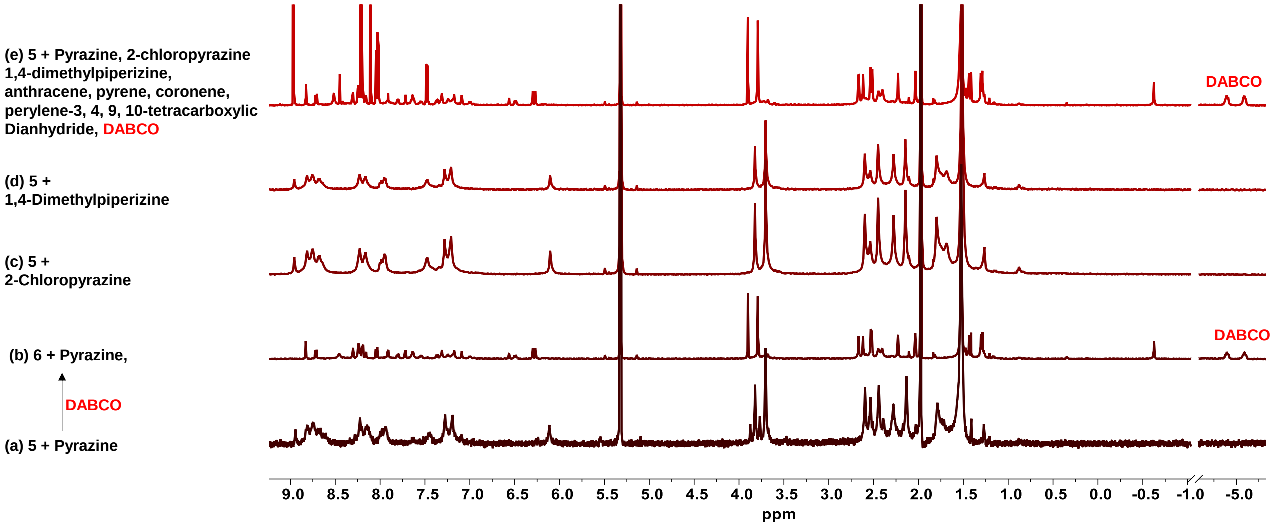

Figure S2. ^{1}H NMR (400 MHz, 298 K) of (a) complex **5** and pyrazine in a ratio of 1:1; (b) complex **5**, pyrazine and DABCO in a ratio of 1:1:1, furnishing complex **6** and leaving the pyrazine free; (c) complex **5** and 2-chloropyrazine in a ratio of 1:1; (d) complex **5** and 1,4-dimethylpiperizine in a ratio of 1:1; (e) complex **5**, pyrazine, 2-chloropyrazine, 1,4-dimethylpiperizine, anthracene, pyrene, coronene, perylene-3,4,9,10-tetracarboxylic dianhydride and DABCO in a ratio of 1:1:1:1:1:1:1:1:1 furnishing complex **6** and free ligands.

7. NMR spectra: ^{1}H NMR, ^{13}C NMR, ^{1}H,^{1}H COSY

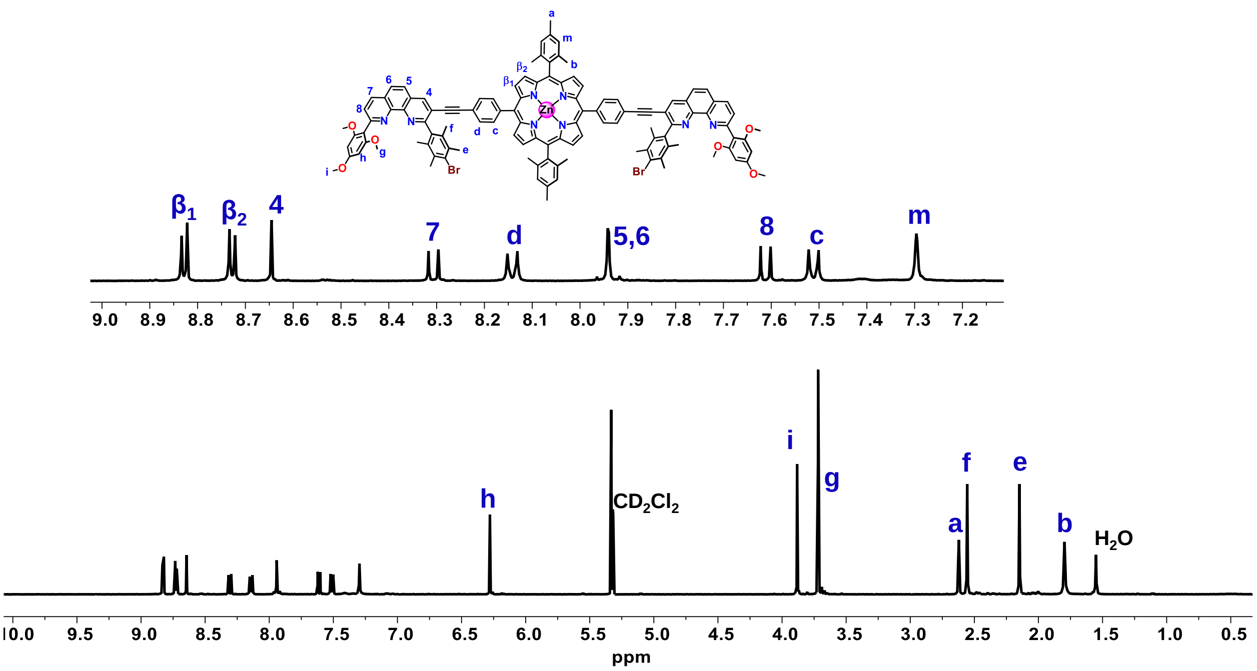

Figure S3. ^{1}H NMR spectrum of **1** in CD_2Cl_2 (400 MHz, 298 K).

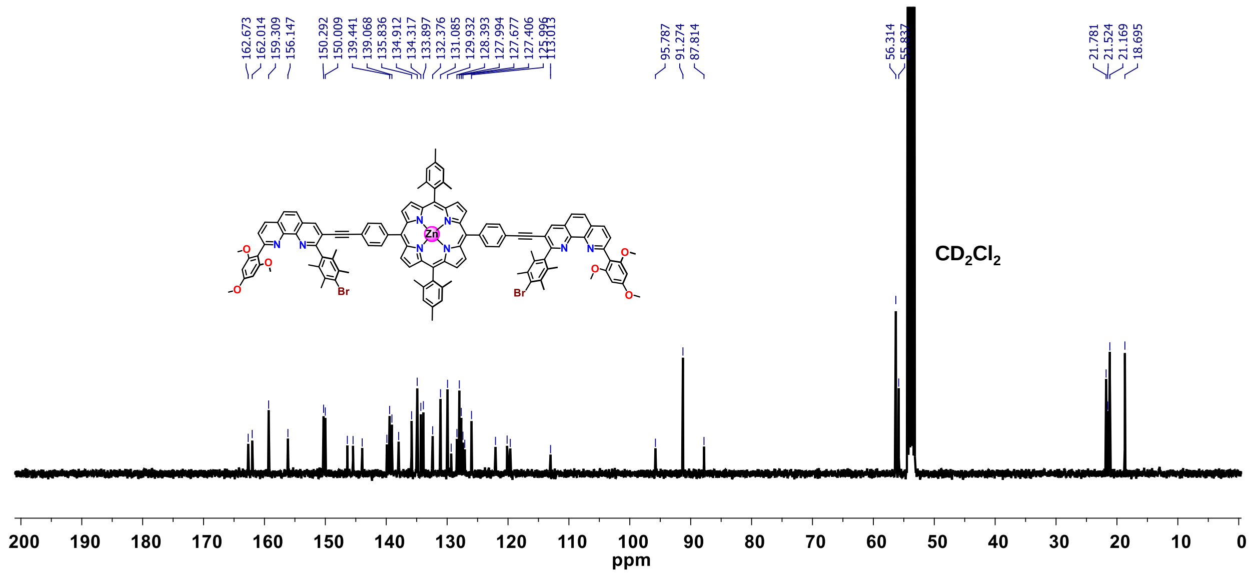

Figure S4. ^{13}C NMR spectrum of **1** in CD_2Cl_2 (100 MHz, 298 K).

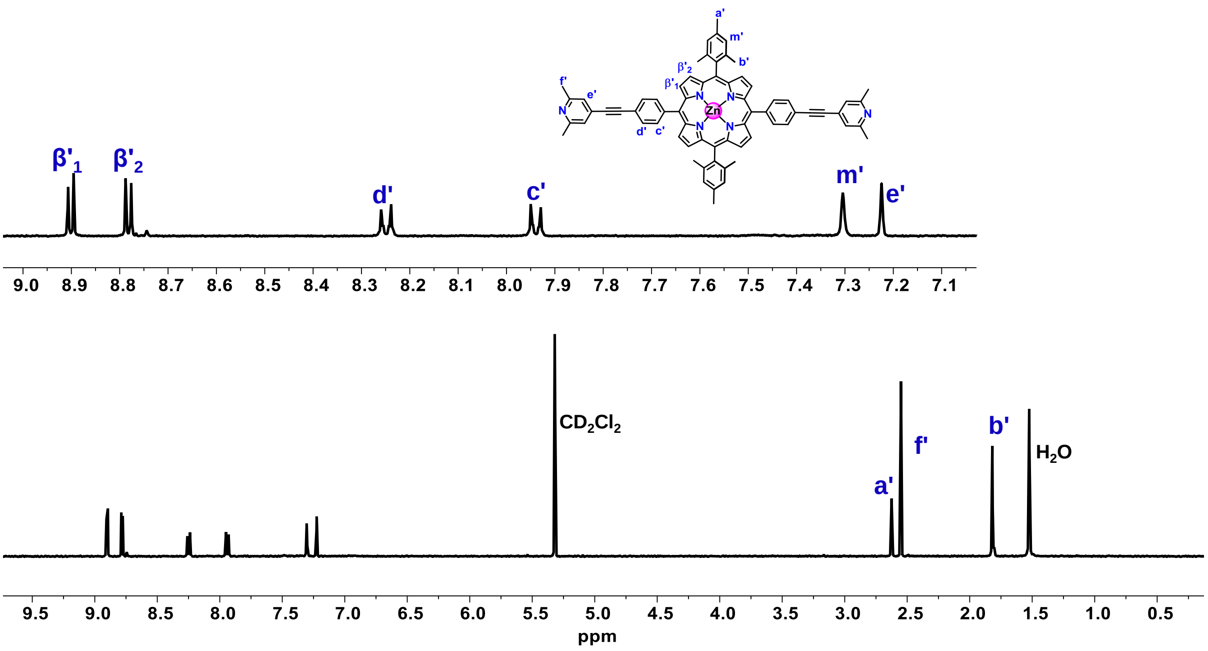

Figure S5. ^{1}H NMR spectrum of **2** in CD_2Cl_2 (400 MHz, 298 K).

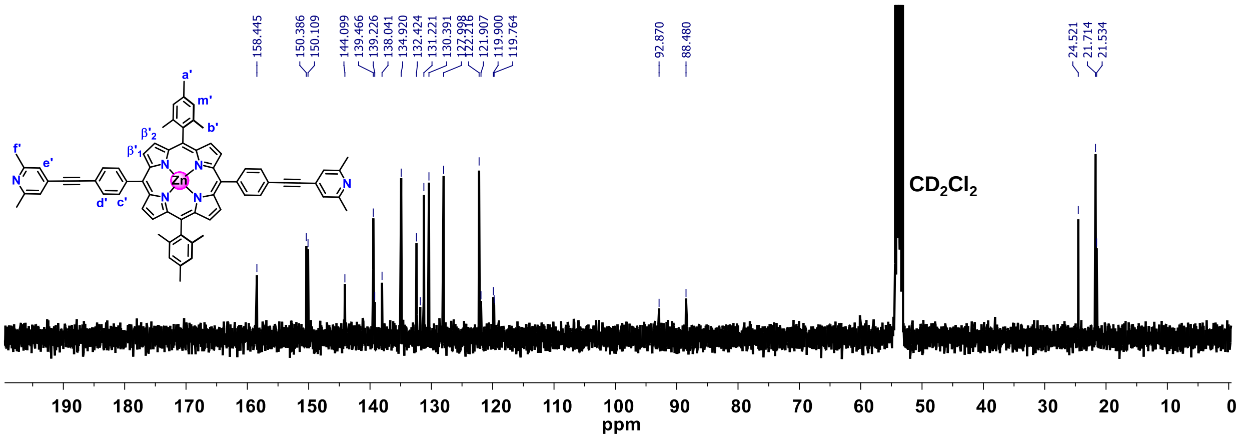

Figure S6. ^{13}C NMR spectrum of **2** in CD_2Cl_2 (100 MHz, 298 K).

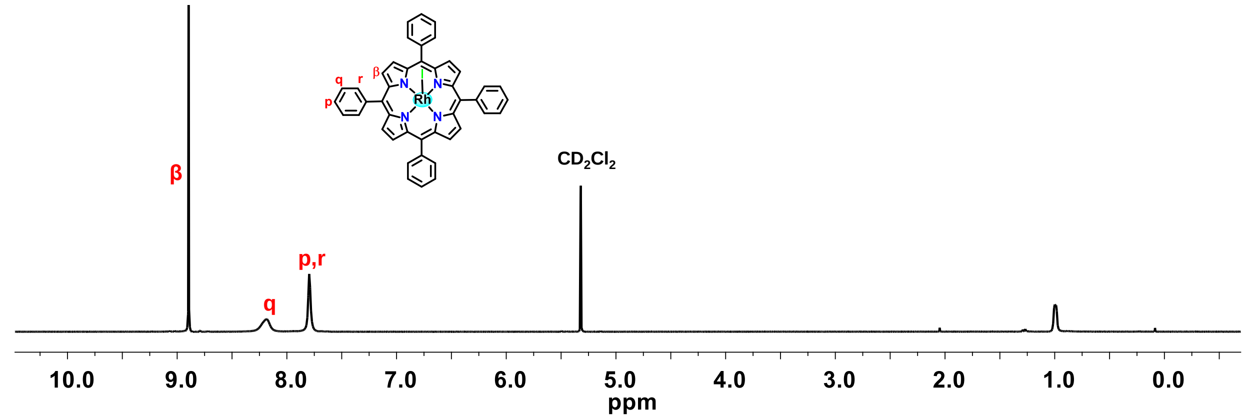

Figure S7. ^{1}H NMR spectrum of **3** in CD_2Cl_2 (400 MHz, 298 K).

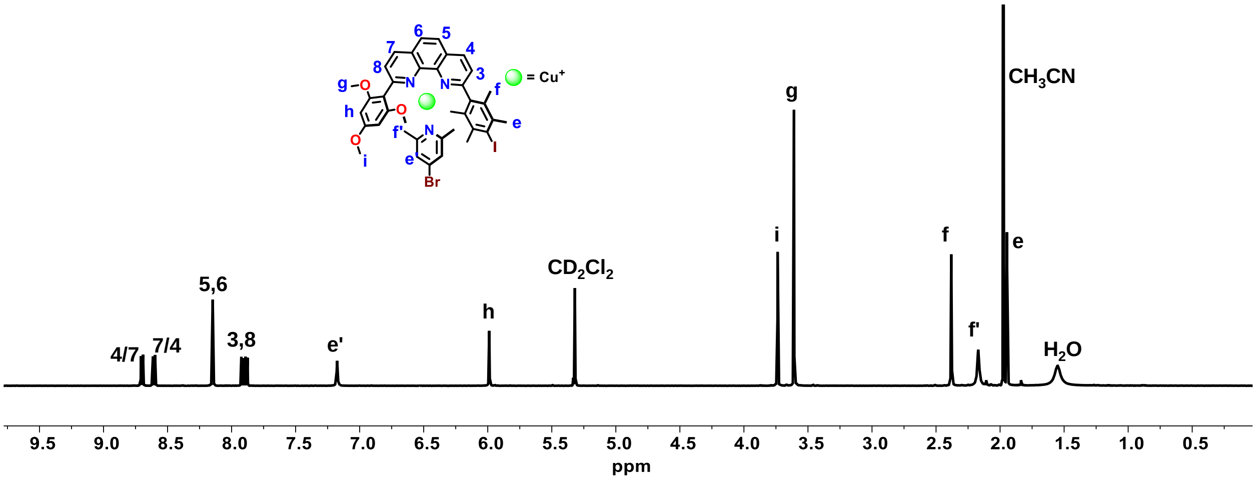

Figure S8. ^{1}H NMR spectrum of complex **11** = $[Cu(\mathbf{8})(\mathbf{9})]^{+}$ in CD_2Cl_2 (400 MHz, 298 K).

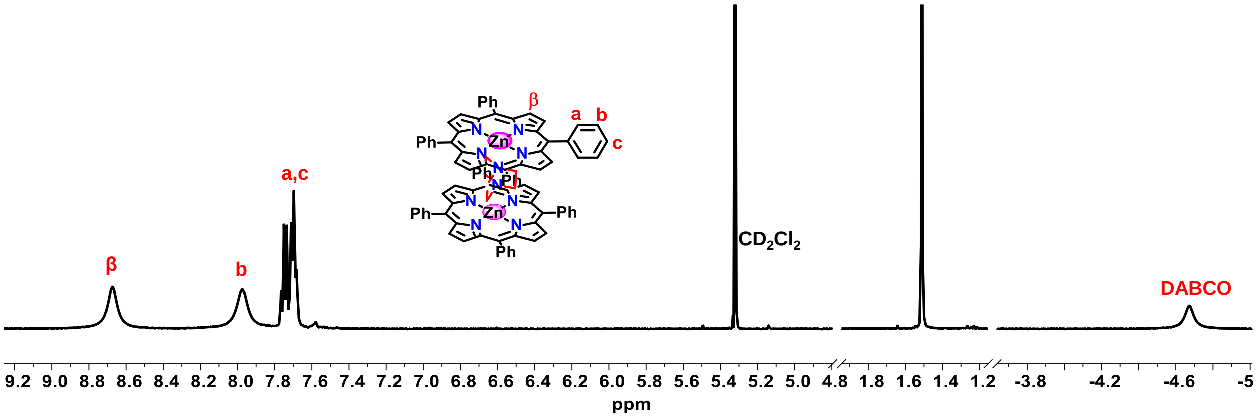

Figure S9. ^{1}H NMR spectrum of complex **12** = $[(\mathbf{4})(\mathbf{10})_3]$ in CD_2Cl_2 (400 MHz, 298 K).

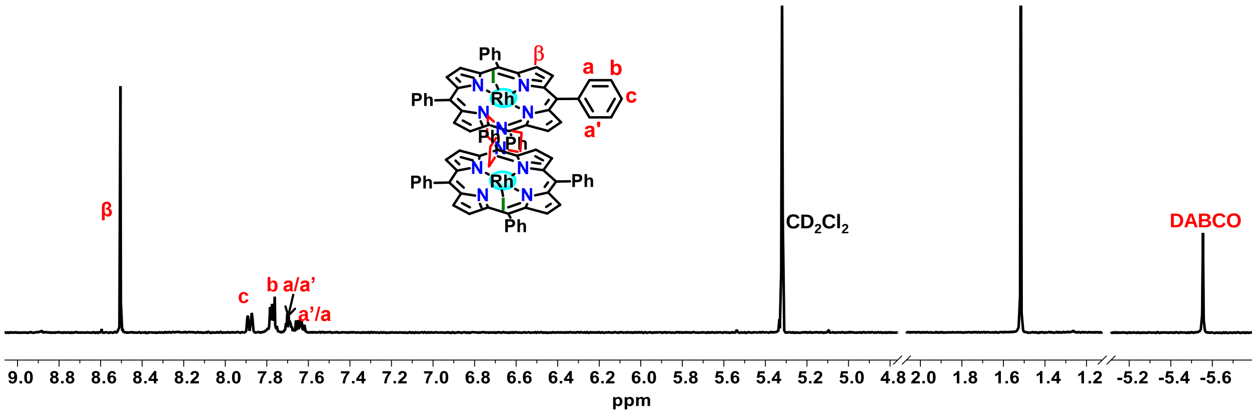

Figure S10. ^{1}H NMR spectrum of complex **7** = [(**3**)$_2$(**4**)] in CD_2Cl_2 (400 MHz, 298 K).

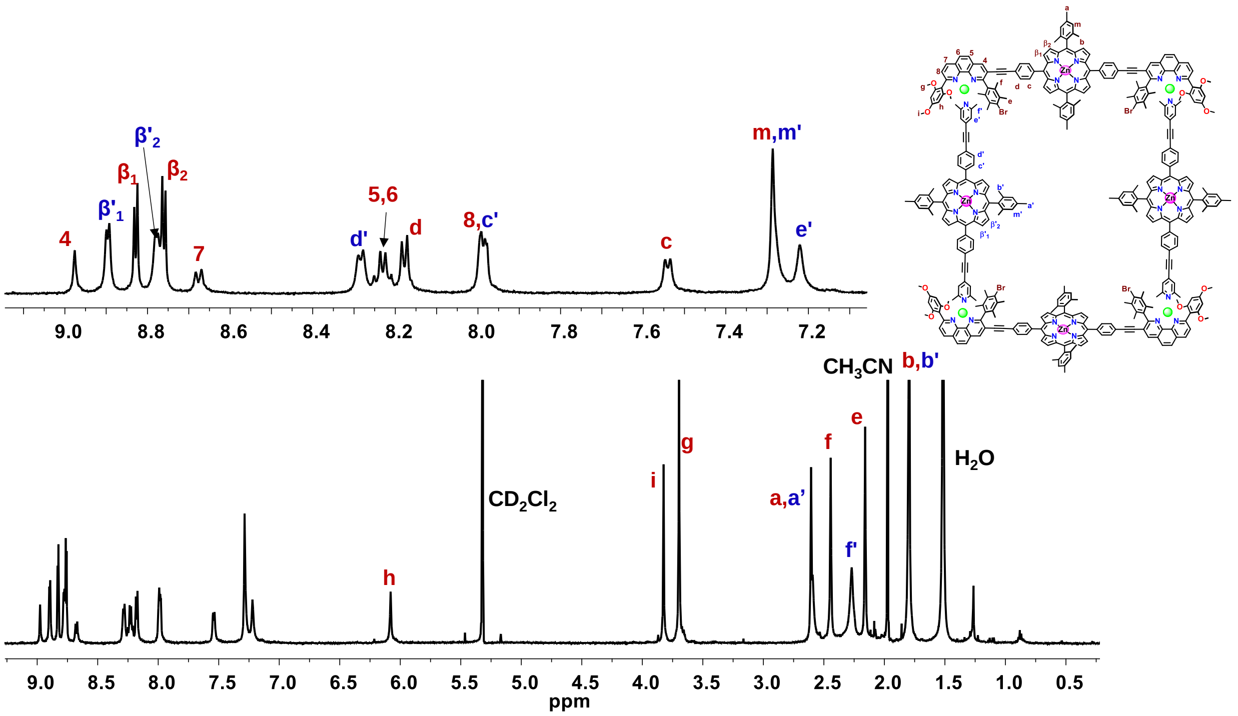

Figure S11. ^{1}H NMR spectrum of complex **5** = [Cu_4(**1**)$_2$(**2**)$_2$]$^{4+}$ in CD_2Cl_2 (400 MHz, 298 K).

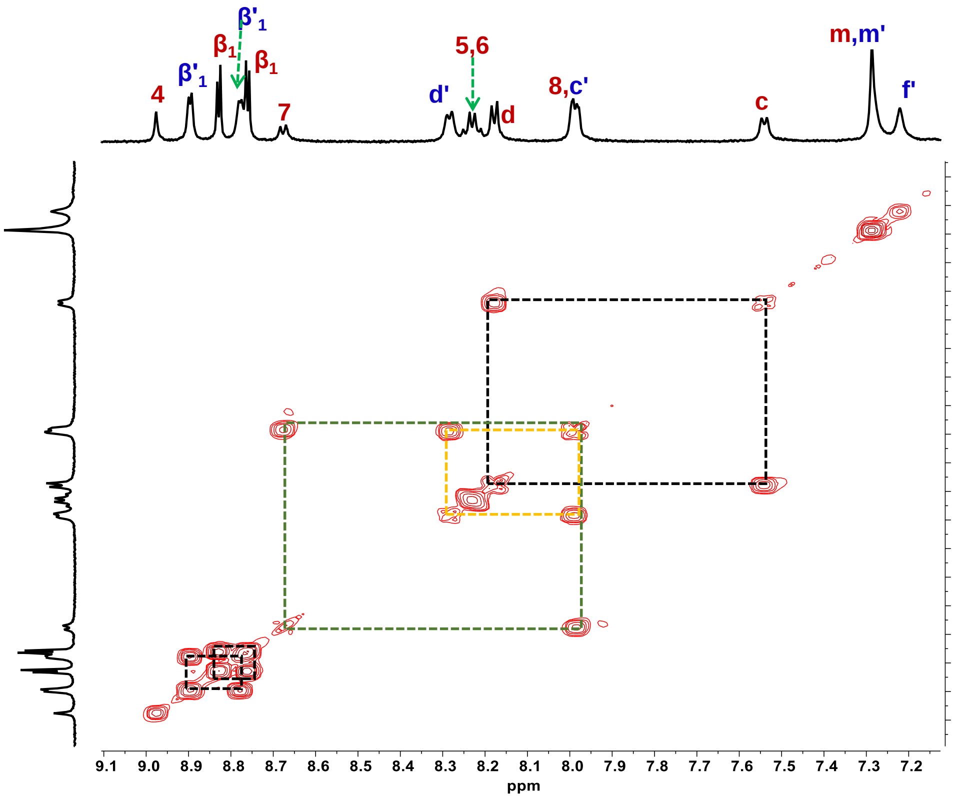

Figure S12. $^{1}H,^{1}H$ COSY spectrum of complex **5** = $[Cu_4(\mathbf{1})_2(\mathbf{2})_2]^{4+}$ in CD_2Cl_2 (400 MHz, 298 K).

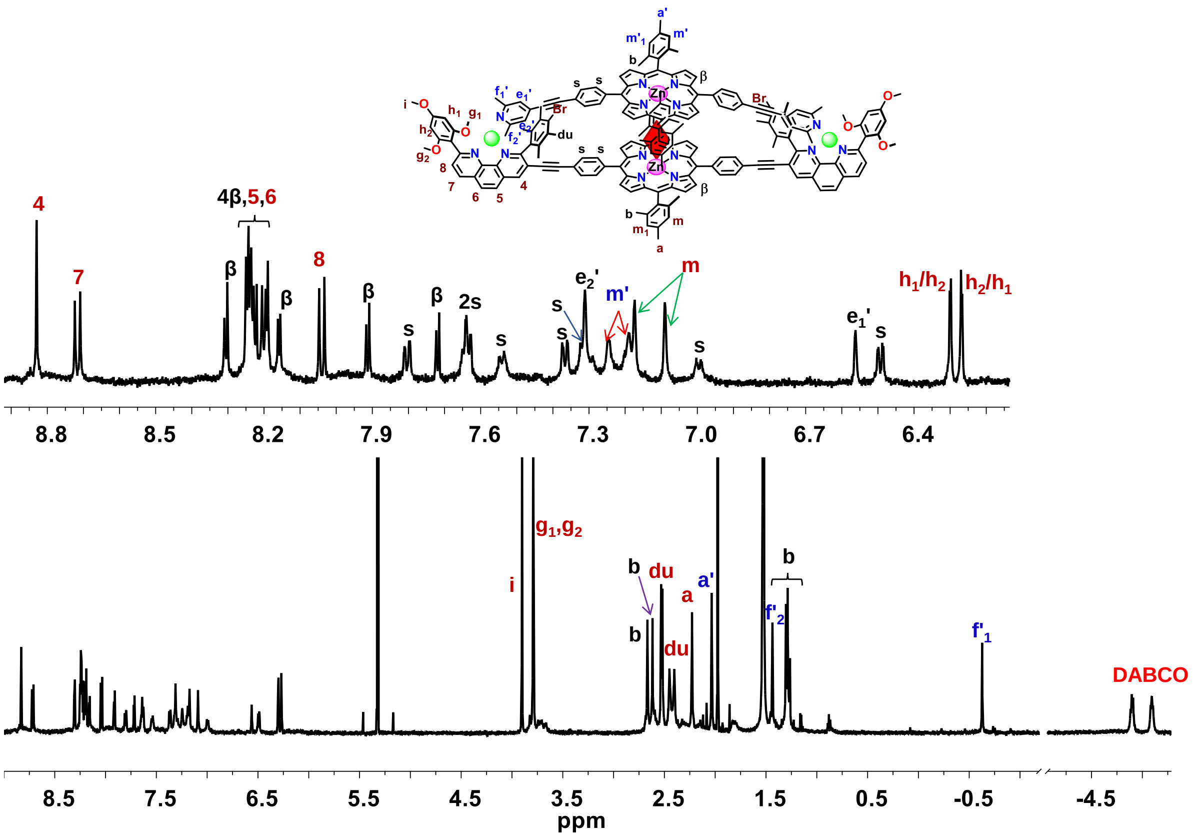

Figure S13. ^{1}H NMR spectrum of complex **6** = $[Cu_2(\mathbf{1})(\mathbf{2})DABCO]^{2+}$ in CD_2Cl_2 (400 MHz, 298 K).

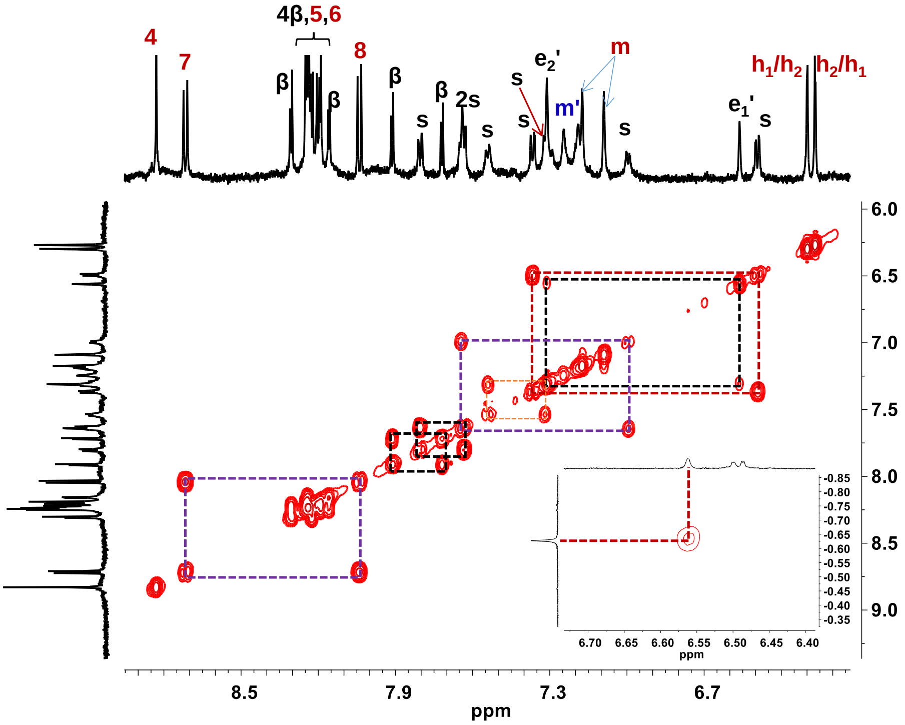

Figure S14. ^{1}H,^{1}H COSY spectrum of complex **6** = $[Cu_2(\mathbf{1})(\mathbf{2})(\mathbf{4})]^{2+}$ in CD_2Cl_2 (400 MHz, 298 K).

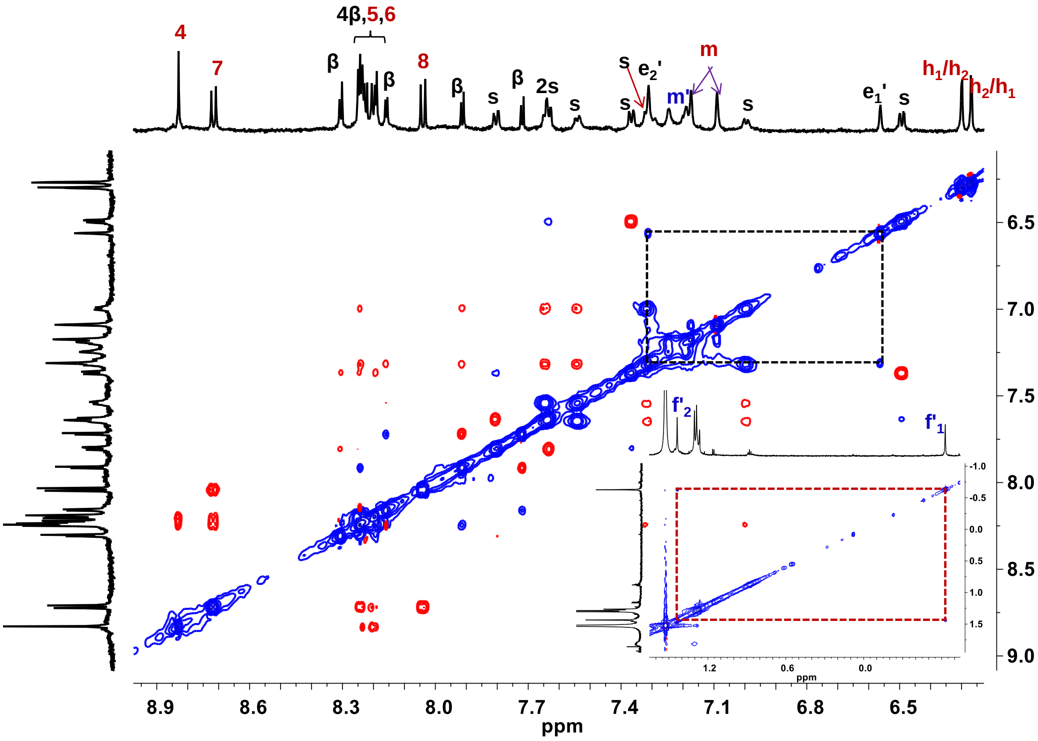

Figure S15. ^{1}H,^{1}H ROESY spectrum of complex **6** = $[Cu_2(\mathbf{1})(\mathbf{2})(\mathbf{4})]^{2+}$ in CD_2Cl_2 (400 MHz, 298 K).

8. ESIMS spectra

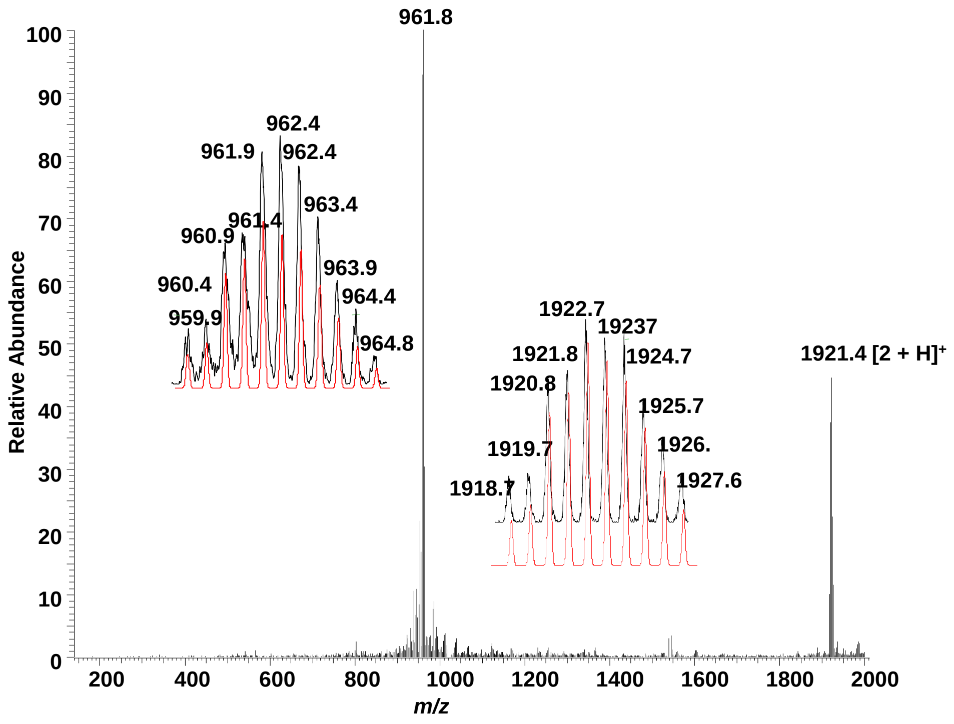

Figure S16. ESI-MS of **1** after protonation.

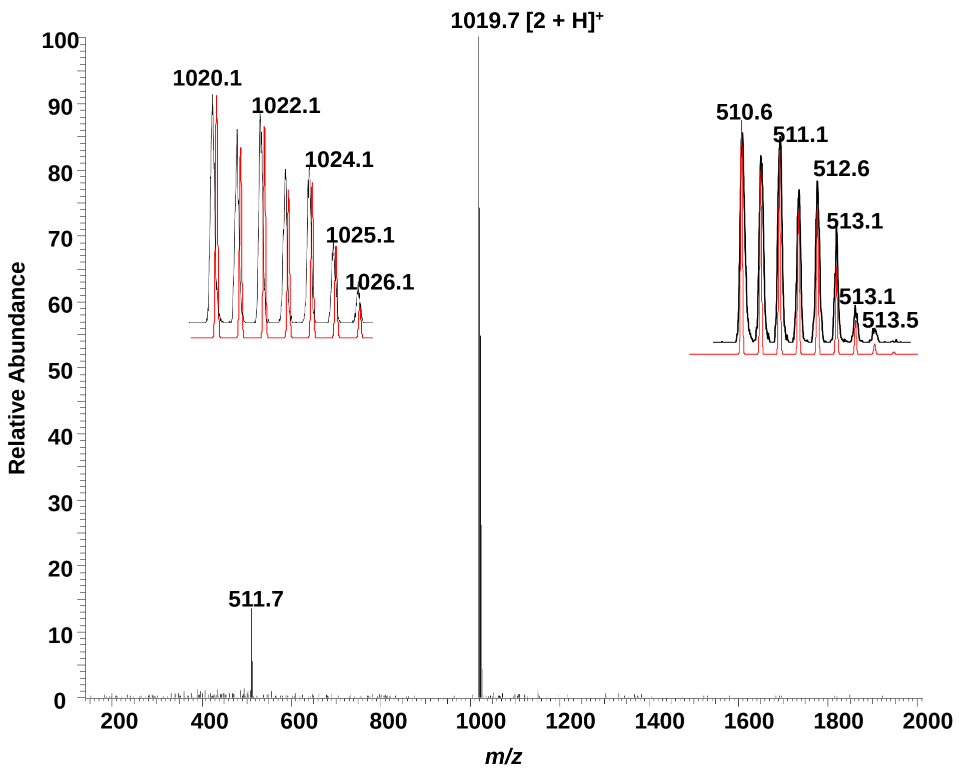

Figure S17. ESIMS of **2** after protonation.

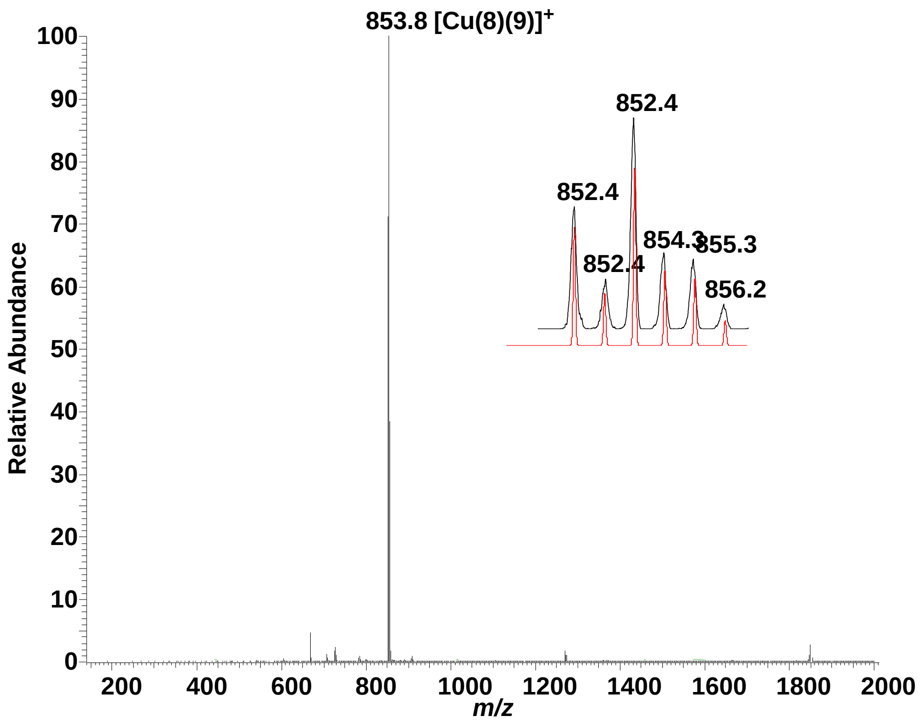

Figure S18. ESIMS of complex **11**.

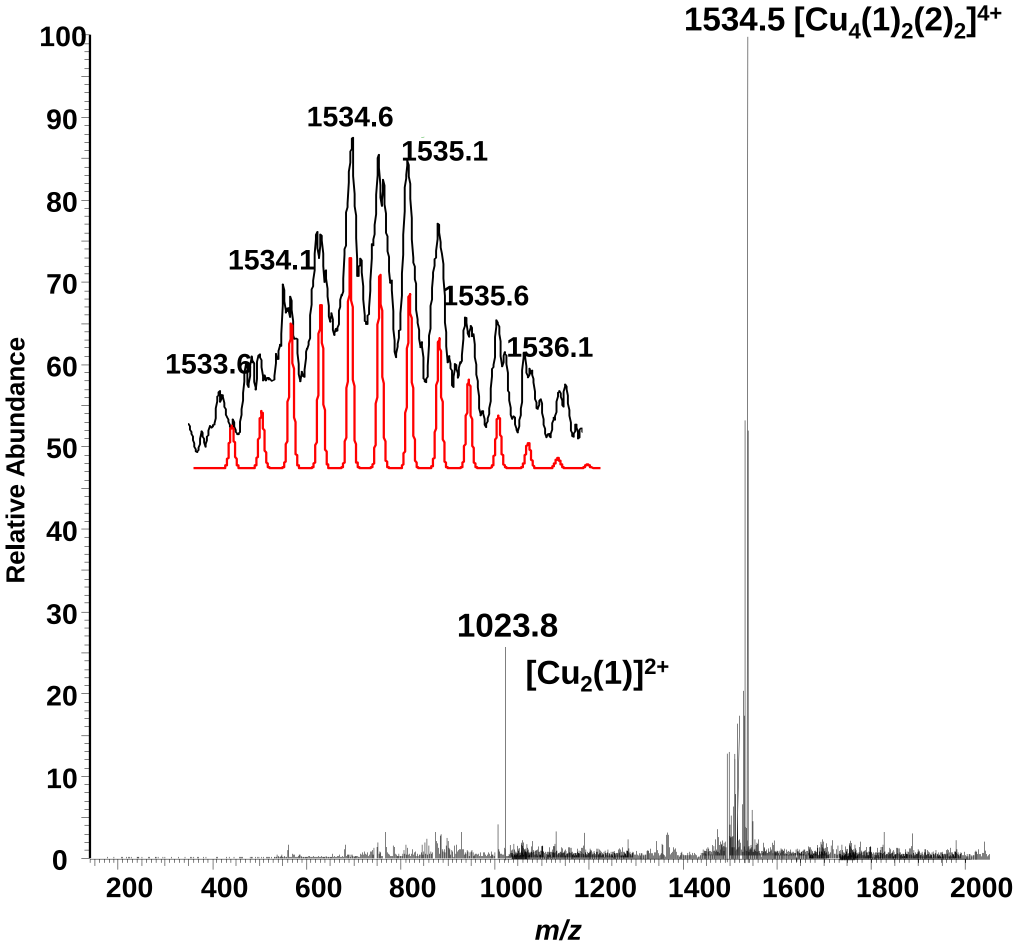

Figure S19. ESIMS of complex **5** = $[Cu_4(\mathbf{1})_2(\mathbf{2})_2]^{4+}$.

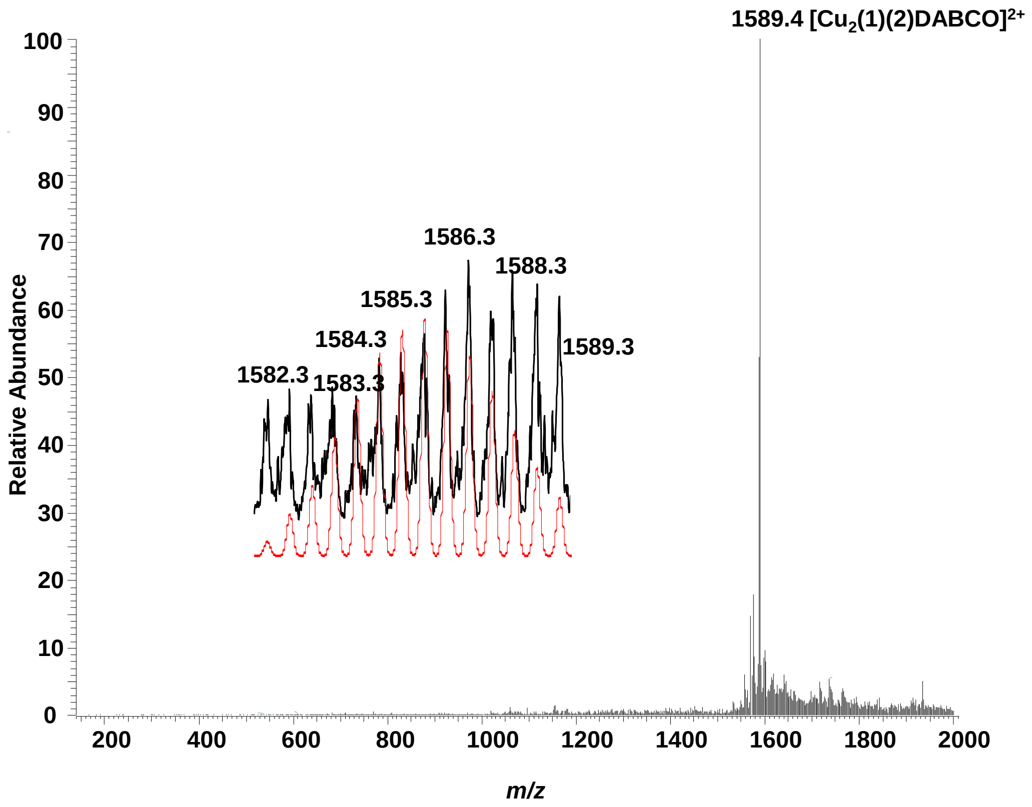

Figure S20. ESIMS of complex **6** = $[Cu_2(\mathbf{1})(\mathbf{2})(\mathbf{4})]^{2+}$.

9. UV–vis data

Measurement of binding constants

The UV–vis titration technique was used to determine the binding constants of complexes. The full data of a selected wavelength region was analyzed using the SPECFIT/32 global analysis system (Spectrum Software Associates, Marlborough, MA).

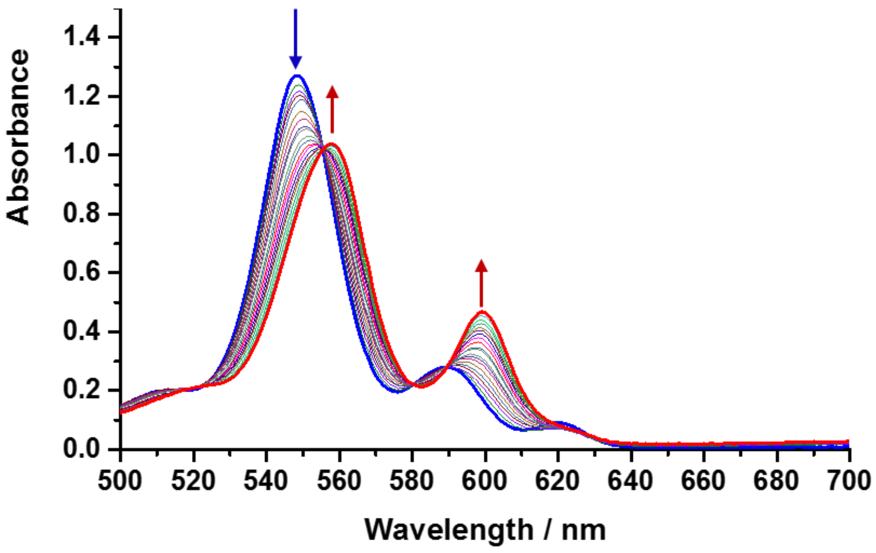

Figure S21. UV–vis spectra of **10** (1.15×10^{-5} M) in CH_2Cl_2 (2 mL) upon addition of DABCO (**4**) (1.10×10^{-4} M) in CH_2Cl_2 at 298 K to afford the complex **12** = [DABCO(**10**)$_2$]. The wavelength region 500–700 nm was analyzed. Result for [DABCO(**10**)$_2$]: log β = 7.20 ± 0.15.

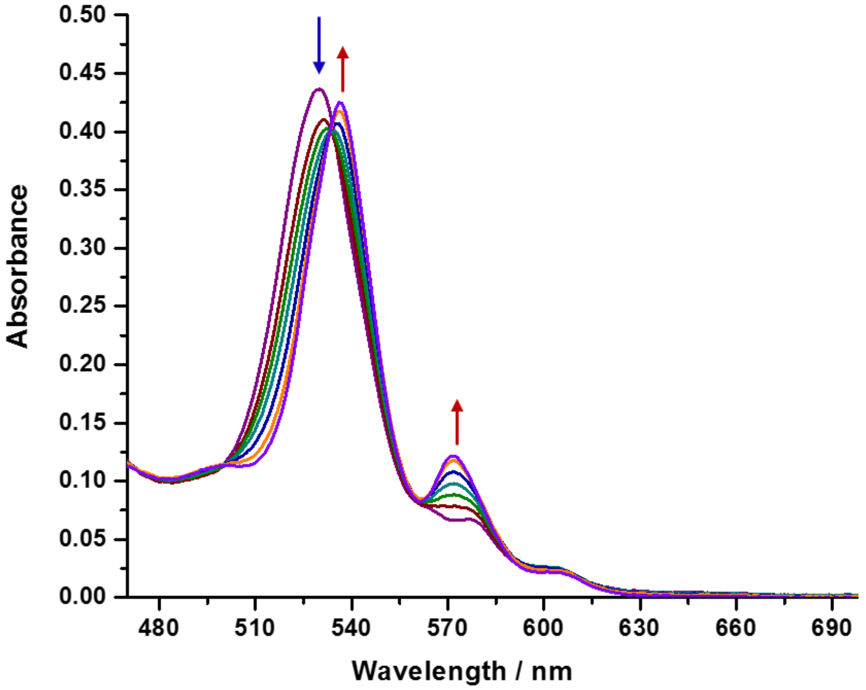

Figure S22. UV–vis spectra of **3** (5.10×10^{-6} M) in CH_2Cl_2 (2 mL) upon addition of DABCO (**4**) (4.08×10^{-4} M) in CH_2Cl_2 at 298 K to afford the complex **7** = [DABCO(**3**)$_2$]. The wavelength region 450–700 nm was analyzed. Result for [DABCO(**3**)$_2$]: log β = 9.60 ± 0.35.

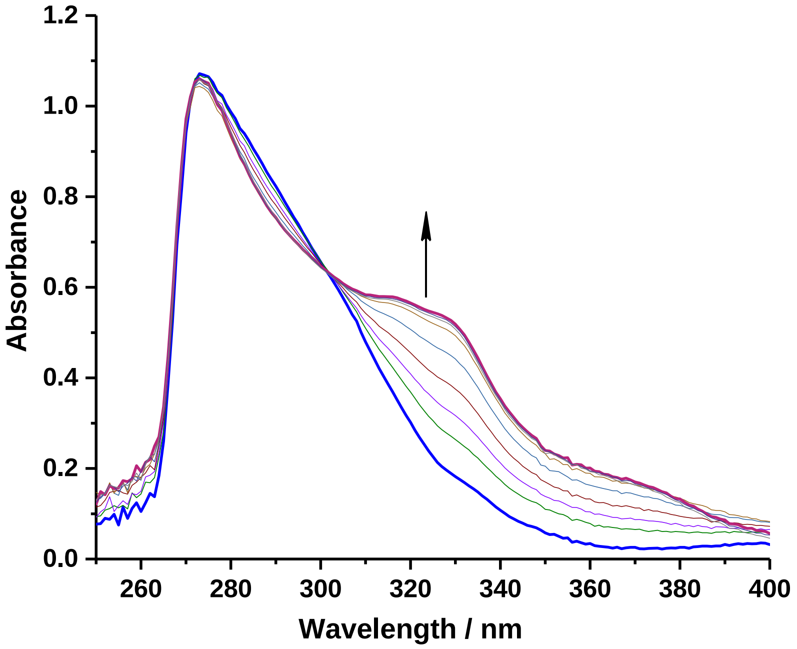

Figure S23. UV–vis spectra of **8** (3.20×10^{-5} M) in CH_2Cl_2 (2 mL) upon addition of $[Cu(CH_3CN)_4]PF_6$ (5.30×10^{-3} M) in CH_3CN at 298 K to afford the complex $[Cu(\mathbf{8})]^+$. The wavelength region 200–400 nm was analyzed. Result for $[Cu(\mathbf{8})]^+$: log $K = 5.64 \pm 0.11$.

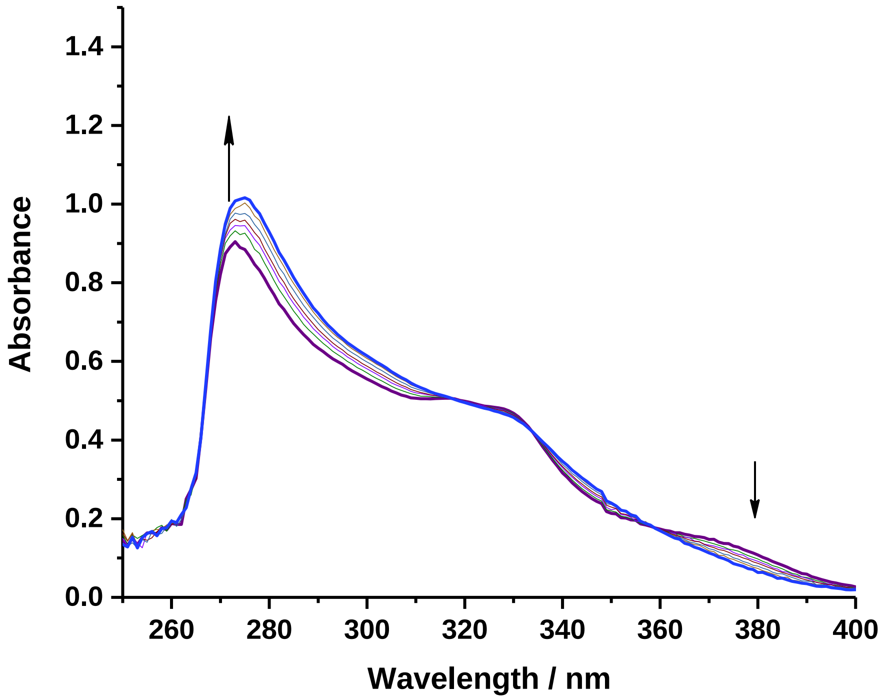

Figure S24. UV–vis spectra of $[Cu(\mathbf{8})]^+$ (3.10×10^{-5} M) in CH_2Cl_2 (2 mL) upon addition of **9** (4.15×10^{-3} M) in CH_2Cl_2 at 298 K to afford the complex $[Cu(\mathbf{8})(\mathbf{9})]^+$. The wavelength region 200–400 nm was analyzed. Result for $[Cu(\mathbf{8})(\mathbf{9})]^+$: log $K = 4.60 \pm 0.21$.

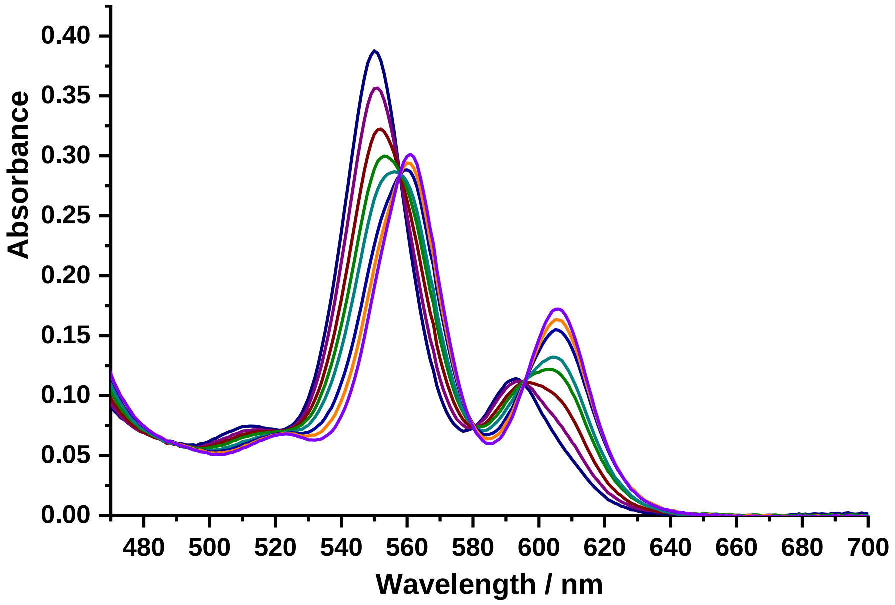

Figure S25. UV–vis spectra of rectangle **5** (4.50×10^{-6} M) in CH_2Cl_2 (2 mL) upon addition of DABCO (**4**) (4.50×10^{-4} M) in CH_2Cl_2 at 298 K to afford the complex **6**. The wavelength region 470–700 nm was analyzed.

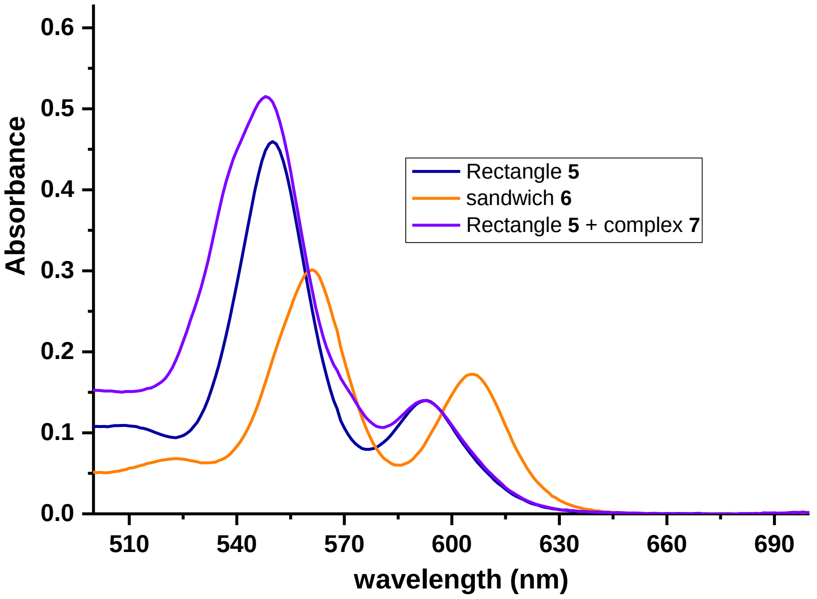

Figure S26. UV–vis analysis of the reversible interconversion between the structure **5** (4.70×10^{-6} M) and **6** (4.70×10^{-6} M) by addition/removal of guest DABCO (**4**) by porphyrin **3** (2.50×10^{-3} M).

10. Fluorescence data

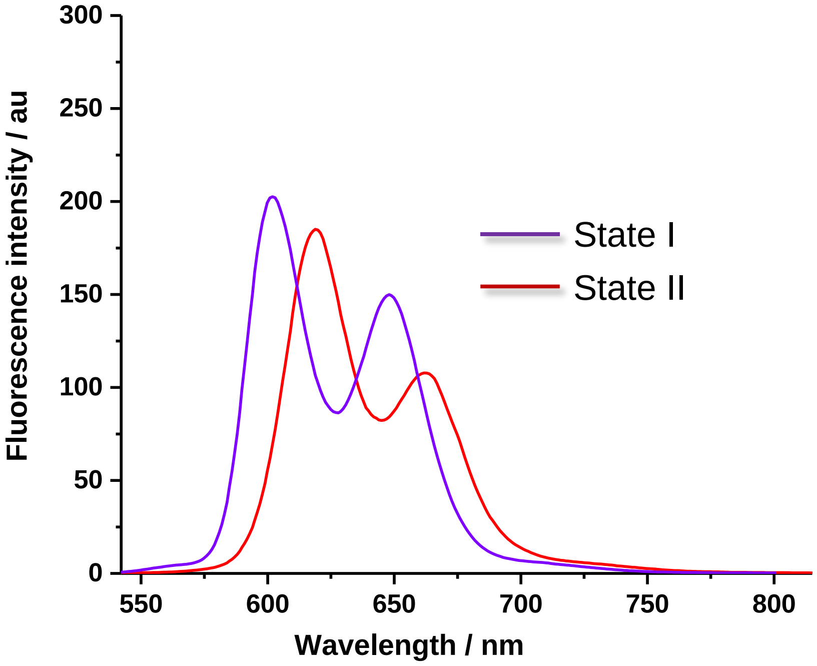

Figure S27. Emission spectra of state I (complex **5**) and state II (complex **6**) (λ_{ex} = 557 nm).

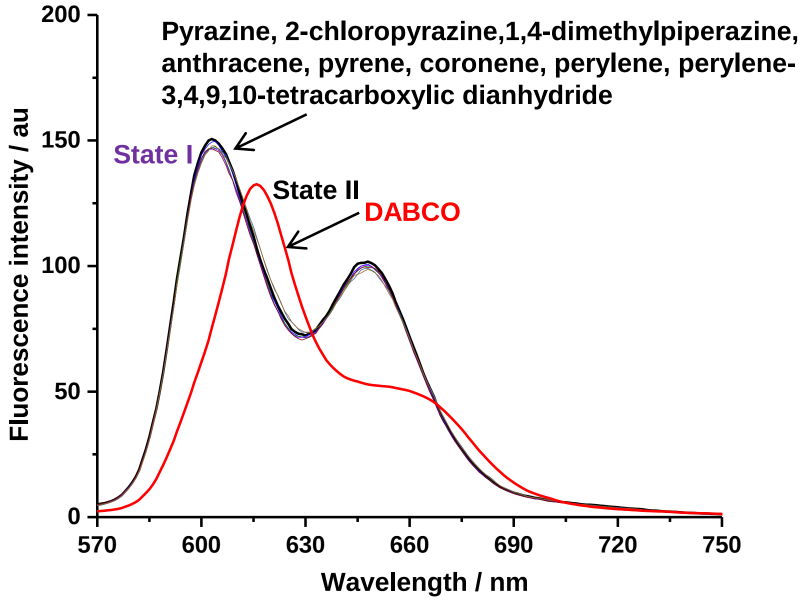

Figure S28. Emission spectroscopy (λ_{ex} = 557 nm) was used to analyze the guest selectivity. All listed guests were sequentially added to complex **5** (state I). In absence of added DABCO, no change in the emission was noted. Even the fluorescence intensity did not change. When lastly DABCO was added the fluorescence spectrum of state II (complex **6**) was generated as indicated by the shift of the emission wavelength to 618 nm.

11. DOSY NMR

Calculation of hydrodynamic radius from

a) The diffusion coefficient D (of complex **5** and **6**) was obtained from the DOSY spectrum and the corresponding hydrodynamic radius was calculated from the Stokes–Einstein equation.

$$r = k_B T/6\eta\pi D$$

b) Radius of the architectures were calculated from their optimized structures at B3LYP/6-31G(d) level.

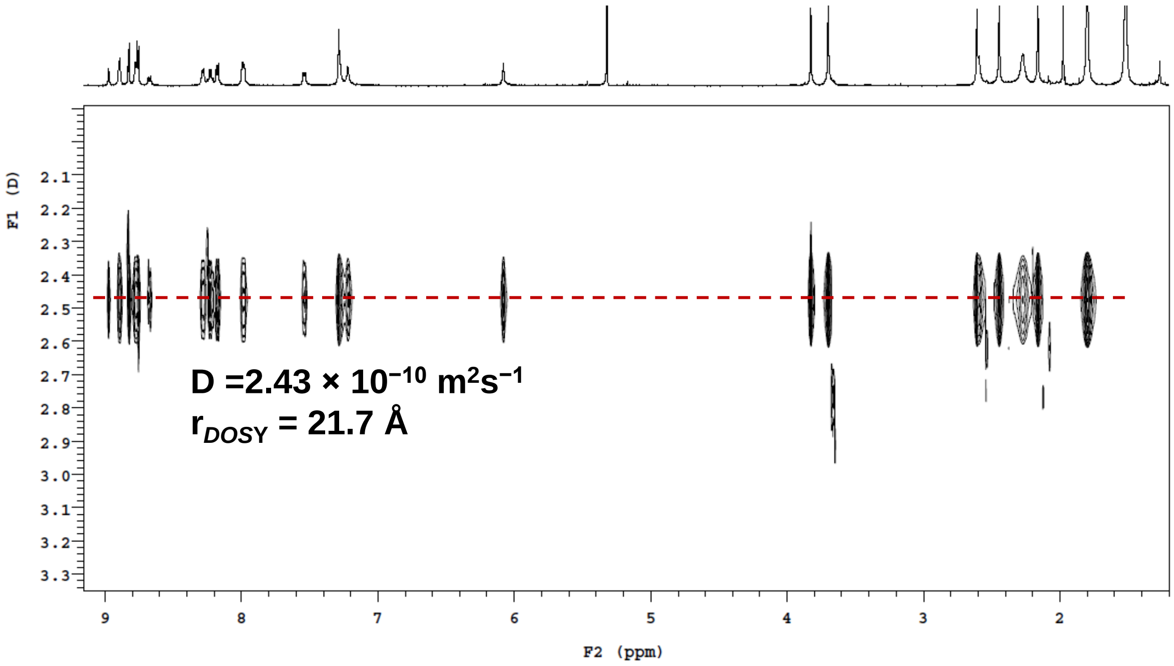

Figure S29. DOSY NMR spectrum (600 MHz, CD_2Cl_2, 298 K) of rectangle **5** = $[Cu_4(\mathbf{1})_2(\mathbf{2})_2](PF_6)_4$.

/

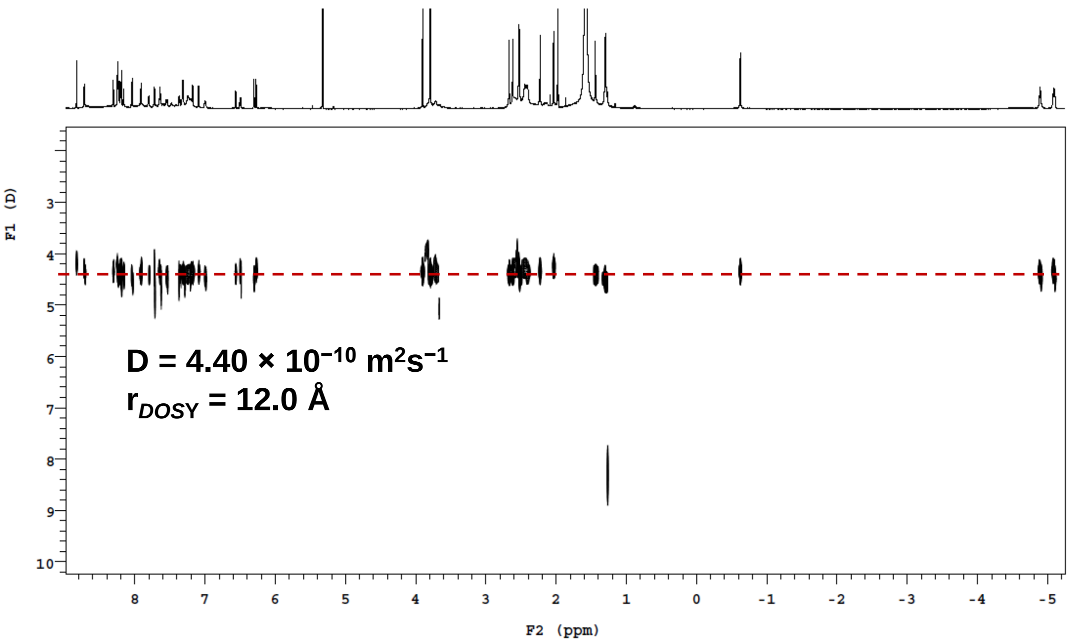

Figure S30. DOSY NMR spectrum (600 MHz, CD_2Cl_2, 298 K) of complex **6** = $[Cu_2(\mathbf{1})(\mathbf{2})DABCO](PF_6)_2$.

12. Calculated structures

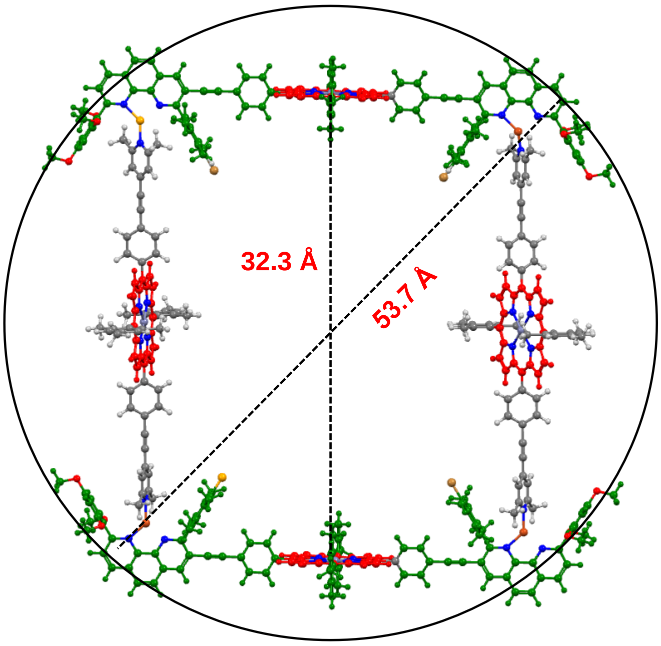

Figure S31. DFT-optimized (B3LYP/6-31G(d)) structure of rectangle **5.** The calculated hydrodynamic radius is 21.5Å.

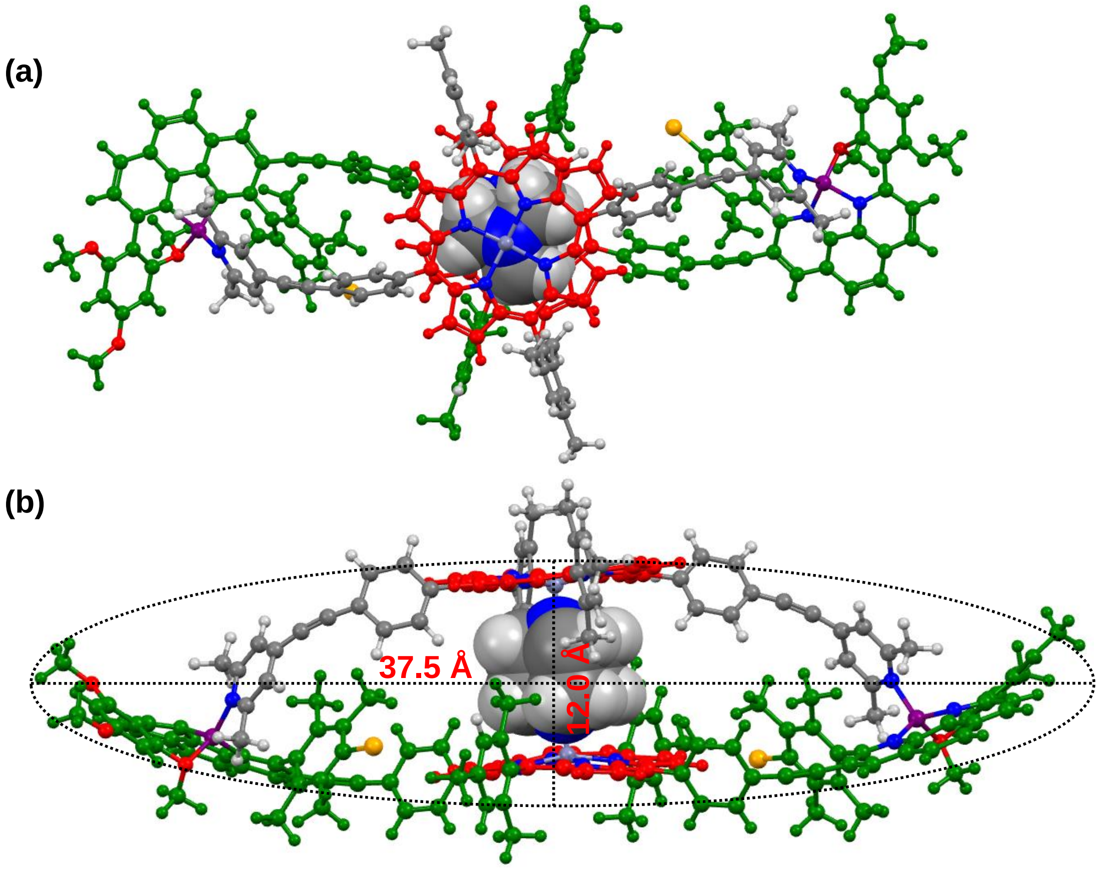

Figure 32. (a) DFT-optimized (B3LYP/6-31G(d)) structure of complex **6** (top view). (b) Side view and calculated hydrodynamic radius from the corresponding structure is 12.3 Å.

13. References

1. Goswami, A.; Paul, I.; Schmittel, M. *Chem. Commun.* **2017**, *53*, 5186.
2. Biswas, P. K.; Saha, S.; Nanaji. Y.; Rana, A.; Schmittel, M. *Inorg. Chem*. **2017**, *56* , 6662
3. Winkelhaus, D.; Neumann, B.; Stammler, H-G.; Mitze, N. W. *Dalton Trans*. **2012**, *41*, 9143.
4. Collman J. P.; Boulatov, R. *J. Am. Chem. Soc*. **2000**, *122*, 11812.
